# Real‐world outcomes of rapid regional hepatitis C virus treatment scale‐up among people who inject drugs in Tayside, Scotland

**DOI:** 10.1111/apt.16728

**Published:** 2021-12-08

**Authors:** Christopher J. Byrne, Lewis Beer, Sarah K. Inglis, Emma Robinson, Andrew Radley, David J. Goldberg, Matthew Hickman, Sharon Hutchinson, John F. Dillon

**Affiliations:** ^1^ Division of Molecular and Clinical Medicine University of Dundee School of Medicine Ninewells Hospital Dundee UK; ^2^ Tayside Clinical Trials Unit University of Dundee Dundee UK; ^3^ Department of Gastroenterology Ninewells Hospital & Medical School Dundee UK; ^4^ Directorate of Public Health National Health Service Tayside Dundee UK; ^5^ Public Health Scotland Glasgow UK; ^6^ School of Health and Life Sciences Glasgow Caledonian University Glasgow UK; ^7^ Population Health Sciences, Bristol Medical School University of Bristol Bristol UK

**Keywords:** direct acting antivirals, elimination, hepatitis C virus, people who inject drugs

## Abstract

**Background:**

In 2017, Tayside, a region in the East of Scotland, rapidly scaled‐up Hepatitis C Virus (HCV) outreach and treatment among People Who Inject Drugs (PWID) using novel community care pathways.

**Aims:**

We aimed to determine treatment outcomes for PWID during the scale‐up against pre‐determined targets; and assess re‐infection, mortality, and post‐treatment follow up.

**Methods:**

HCV treatment was delivered in community pharmacies, drug treatment centres, nurse‐led outreach clinics, prisons, and needle exchanges, alongside conventional hospital care. We retrospectively analysed clinical outcomes and compared pathways using logistic regression models.

**Results:**

Of 800 estimated HCV‐infected PWID, 718 (90%) were diagnosed. 713 treatments commenced among 662 (92%) PWID, delivering 577 (81%) Sustained Virologic Responses (SVR). SVR was 91% among those who attended for testing. Forty‐six individuals were treated more than once. Needle exchanges and community pharmacies initiated 49% of all treatments. Regression analyses implied pharmacies had superior follow‐up, but there was no difference in likelihood of achieving SVR in community pathways relative to hospital care. Re‐infection occurred 39 times over 256.57 person years (PY), yielding a rate of 15.20 per 100 PY (95% CI 10.81‐20.78). 54 deaths occurred (29 drug related) over 1,553.04 PY, yielding a mortality rate of 3.48 per 100 PY (95% CI 2.61‐4.54). Drug‐related mortality was 1.87 per 100 PY (95% CI 1.25‐2.68).

**Conclusions:**

Rapid HCV treatment scale‐up to PWID in community settings, whilst maintaining high SVR, is achievable. However, other interventions are required to minimise re‐infection; reduce drug‐related deaths; and improve post‐SVR follow‐up testing regionally.

## Graphical abstract

Rapid regional HCV treatment scale‐up was achieved in Tayside. There were over 700 treatments across six pathways in three years, a close to two‐fold increase on previously high levels of HCV treatment among PWID. The estimated care cascade shows that 90% of the estimated HCV‐infected PWID population were diagnosed and 92% of those initiated treatment. SVR in the ITT group was over 80% and exceeded 90% in those with a documented test.

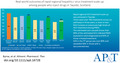

## 1 INTRODUCTION

Hepatitis C Virus (HCV) is a blood‐borne virus (BBV) that is transmitted mainly via percutaneous exposure to infected blood. Therapeutic advances led the World Health Organization, in 2016, to release its Global Health Sector Strategy, providing an implementation plan for HCV elimination.^1^ It specified targets to diagnose 90% of infected individuals and initiate treatment for 80% of those diagnosed, to facilitate HCV elimination by 2030.

In 2017, concurrent to the WHO strategy response, National Health Service (NHS) Tayside—a health board in the East of Scotland serving approximately 416,000 people—commenced a trial of Treatment as Prevention (TasP) in HCV infection, by rapidly scaling up treatment among PWID.^2^, ^3^ The first step was a regional programme of intensified HCV testing and treatment to reduce chronic HCV to <10% among PWID over the long term and achieve WHO elimination criteria.^3^ The concept of TasP is using treatment to lower HCV prevalence among PWID to prevent new infections and re‐infections, maintaining elimination or reducing the amount of treatment needed to maintain it.^3^, ^4^

The programme was underpinned by novel community care pathways, and highly effective Direct Acting Antiviral (DAA) treatments.^5^, ^6^ The pathways were implemented by a multidisciplinary team. Clinical initiatives focused on PWID, as injection drug use (IDU) was the primary driver of regional HCV transmission, and over 90% of HCV infections in Scotland occur consequent to IDU.^7^, ^8^

This study aims to determine if rapid regional scaleup of HCV treatment was achieved in the PWID population in line with local and WHO targets. We report critical real‐world outcomes of the scale‐up including cure rates (Sustained Virologic Response, SVR) by treatment pathway, risks of HCV re‐infection, mortality rates, and effectiveness of post‐treatment follow‐up, both for SVR and re‐infection testing.

## 2 METHODS

### 2.1 Testing and treatment targets

Prior to scaleup, there were an estimated 2800 PWID resident in the Tayside heath board region, of whom 800 (29%) were estimated to have chronic (6+ months infected) HCV.^3^, ^9^ In line with these estimates, local targets were set to achieve the prevalence reduction required to demonstrate TasP over the long term. Targets were to diagnose and engage approximately 680 (85%) HCV infections among PWID, initiate approximately 592 (87% of diagnosed) treatments, and obtain approximately 533 (≥90% of treated cases) undetected SVRs.

### 2.2 Study setup

The study is described on clinicaltrials.gov (NCT03356405) and ISCRCTN (ISRCTN72038467) and co‐sponsored by NHS Tayside and University of Dundee (2016GA08). It received NHS ethical favourable opinion (17/ES/0136). Data was obtained from HCV clinical databases; virology/microbiology records; and patient records. Caldicott Guardian approval for this was granted (IGTCAL7005).^10^ Tracking individuals was by Community Health Index (CHI) number, an identifier allocated to every registered patient in Scotland. All adults who initiated HCV treatment with DAAs from January 2017 to mid‐April 2020, immediately prior to Tayside's elimination declaration, who acquired HCV via IDU, were eligible.^11^ Total number of HCV‐diagnosed PWID includes late‐2016 diagnoses. Individuals self‐reported injecting status to clinical staff. Follow‐up was censored in mid‐December 2020. A de‐identified database was created and stored on secure servers, with participants allocated unique identifiers.

Individual parameters included: CHI; date of birth; gender; and post code of most recent residence to derive Scottish Index of Multiple Deprivation (SIMD).^12^ Clinical data included: dates of HCV diagnoses; number of treatments; transmission route; genotype; treatment pathway; recent IDU; cirrhosis status; BBV co‐infection status; opioid substitution therapy (OST) status; treatment type; treatment start date; treatment completion status; mortality status and, if deceased; mortality cause.

### 2.3 Testing

HCV RNA testing on plasma samples was undertaken using the Hologic Panther platform with HCV Quant Dx Assay^13^ or the Abbott Real‐Time m2000sp and m2000rt Polymerase Chain Reaction (PCR) platform.^14^ HCV genotype analysis, including subtyping, used an in‐house real‐time PCR as the front line assay and, if required, HCV core Sanger sequencing to determine any genotypes which were unclear by real‐time PCR, at the Scottish Virology Reference Laboratory, NHS Greater Glasgow & Clyde. Dried Blood Spot (DBS) RNA samples were tested using an in‐house assay^15^ or the Abbott m2000 system.^16^

### 2.4 Patient and public involvement

Patient or public involvement groups were not involved in the design of this study. Study design was developed from prior experience of the investigators working with the stakeholders involved.

### 2.5 Population and outcome definitions

PWID was defined as ever injected drugs (self‐reported). Cirrhosis was defined as any of the following: Fibroscan reading >18kPa; Fib4 >3.46, or confirmation via: liver biopsy; ascites with evidence of liver disease; hepatic encephalopathy plus chronic liver disease; oesophagogastric varices with patent portal vein. SVR was undetectable HCV RNA at least 12 weeks post‐treatment. Relapse was undetectable RNA at end of treatment, but detectable prior to or at SVR; or treatment initiation and detectable RNA prior to or at SVR, if end of treatment test not conducted. Non‐response was detectable RNA above the lower limit of detection (10 IU/mL) during treatment. Re‐infection was detectable RNA following confirmed SVR, or detectable RNA at end of treatment with genotype change. Cause of death was classified as: liver related (or not); drug related (in line with National Records Scotland; NRS)^17^ and unknown/other if mortuary record not finalised at censor date, or death occurred outside Tayside. Liver‐related death was liver disease as primary/secondary morality cause on an individual's mortuary record. Although mortality causes could only be obtained for those in Tayside, mortality status was determined via CHI number. OST receipt was defined at the time of pre‐treatment HCV PCR test ±6 months.

### 2.6 Treatment pathways

There are six HCV care pathways in Tayside; five in community settings. They are named by location: conventional hospital care; drug treatment centres; needle exchanges; community pharmacies; nurse‐led outreach clinics and prisons. All community pathways are in environments accessible to PWID. In the nurse‐led outreach pathway, patients are seen in their local neighbourhood, typically in remote areas. Drug treatment clinics are in central locations. Testing, pre‐treatment assessment, and treatment are available in each (ultrasound requires central travel). The needle exchange and community pharmacy pathways were initially experimental, implemented through pragmatic pathway trials, which became embedded to provide routine testing and treatment.^18^, ^19^, ^20^, ^21^ All pathways are multidisciplinary, involving specialist nursing staff, gastroenterologists/hepatologists, infectious disease physicians, specialist and community pharmacists, and psychologists. Nurses and community pharmacists can prescribe DAAs for simple cases of HCV infection (eg treatment naïve and non‐cirrhotic), either as independent prescribers or through Patient Group Direction.^22^

### 2.7 Statistical analysis

Primary outcomes (treatments and SVR) were summarised using descriptive statistics, for comparison against targets. Comparison of SVR by patient and treatment characteristics were conducted in the same manner in intention‐to‐treat (ITT) and per‐protocol (PP) populations. The ITT group includes all who initiated treatment, and the PP group includes treatment completers. We hypothesised that variation in SVR in community pathways compared to specialist hospital care would be due to differences in follow‐up. Logistic regression modelling was used to assess this. Models were adjusted for patient characteristics and explored steps in the treatment journey for the ITT and PP groups, with and without SVR tests. Models were adjusted in a stepwise approach for age, gender, cirrhosis, and genotype. Where applicable, participants missing SVR were assumed treatment failures. *P* of ≤0.05 was assumed to demonstrate statistical significance. Analyses were undertaken using IBM SPSS Statistics 25.

### 2.8 Re‐infection and mortality

Those who achieve SVR are offered annual follow‐up RNA testing through all pathways. We measured the efficacy of re‐infection follow‐up by calculating the proportion of cases with follow‐up RNA tests received in line with local policy (annual re‐testing post‐SVR). We estimated re‐infection incidence per 100 person‐years (PY), assuming a Poisson distribution. Time‐at‐risk began after SVR and ended at last negative RNA test, if not re‐infected. If re‐infected, time‐at‐risk ended at the mid‐point between last negative RNA test and re‐infection date. Where last negative RNA test indicated SVR, with no further follow‐up tests prior to the censor date, cases were excluded. Cases that did not achieve SVR or died prior to this were excluded. For cases that were re‐infected at time of SVR test, that is RNA negative at end‐of‐treatment, or missing end‐of‐treatment test, then RNA positive at SVR with different genotype, the time‐at‐risk period was from the date of end‐of‐treatment RNA test, or estimated date if test not available, and the mid‐point between then and date of SVR. Mortality was checked up to and including the censor date; rates were based on time‐at‐risk from treatment initiation for primary HCV infection to censor date or date of decease.

## 3 RESULTS

**FIGURE 1:**
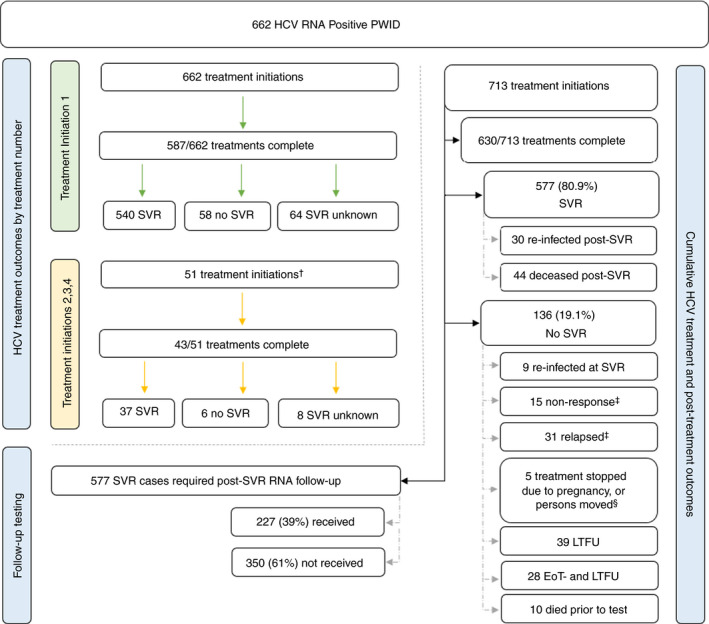
Post‐treatment outcomes of People Who Inject Drugs treated in NHS Tayside 2017‐20. HCV, Hepatitis C Virus; RNA, Ribonucleic acid; PWID, People Who Inject Drugs; SVR, Sustained Virologic Response; LTFU, Lost to Follow‐up; EoT‐ and LTFU, End of Treatment Negative and Lost to Follow‐up. ^†^Five were treated 3 or 4 times. ^‡^For disclosure reasons, some data are combined. ^§^Of relapses and non‐responses, eleven completed treatment. It is possible some cases are early re‐infections, but the evidence was insufficient to classify accordingly

Annual treatments initiations among PWID in Tayside for the 3 years prior to scale‐up (2014‐16) were, 126, 138, and 130, giving a cumulative 394 treatments among 387 PWID. During treatment scaleup (January 2017 to April 2020), 713 treatments were initiated among 662 PWID (Figure 1), an 84.5% increase in HCV treatment among PWID regionally.

Table 1 outlines descriptive cohort data (tabulated by pathway in Data S1). All patients were treated with DAAs. All patients had an injection history, 77.3% received OST and 51.9% reported injecting in the 12 months prior to treatment. Most were male. Median age at first treatment was just under 40. Most individuals had a registered postcode in the most deprived areas. HCV genotypes 1 and 3 were most common. Co‐infection with other BBVs was uncommon, and few were diagnosed with cirrhosis. Approximately one third were treated through a pragmatic trial of a novel pathway.

**TABLE 1:** Descriptive characteristics of PWID treated for HCV in NHS Tayside 2017‐2020

	Characteristic	HCV‐treated PWID
Demographic^a^	Gender—n (%)	
	Male	491 (74.2)
	Female	171 (25.8)
	Postcode Type—n (%)	
	Residential	605 (91.4)
	Supported living or hostel	41 (6.2)
	Prison	16 (2.4)
	SIMD postal code quintile—n (%)	
	(most deprived) 1	348 (52.6)
	2	184 (27.8)
	3	67 (10.1)
	4	51 (7.7)
	(least deprived) 5	8 (1.2)
	Not indexed	4 (0.6)
	Age in years—median (IQR) [n];	
	At first HCV diagnosis	34 (28‐40) [662]
	At first HCV treatment	39 (34‐46) [662]
	At second HCV treatment	40 (36‐47) [46]
	Pre‐scaleup HCV treatment history—n (%)	
	Treatment experienced	119 (18.0)
	Treatment naïve	543 (82.0)
	Pre‐scaleup HCV treatments received—n (%)	
	1	111 (93.3)
	2	8 (6.7)
	Pre‐scaleup HCV treatment type—n (%)	
	Peg‐IFN based	114 (89.8)
	DAA based	13 (10.2)
Pre‐treatment information^b^	HCV Genotype—n (%)	
	1	280 (39.3)
	3	403 (56.5)
	2 or 4	4 (2.0)
	Unknown	16 (2.2)
	Liver cirrhosis indicated pre‐treatment—n (%)	
	Yes	65 (9.1)
	No	646 (90.6)
	Unknown	2 (0.3)
	Report injecting in 12 months prior to treatment—n (%)	
	Yes	370 (51.9)
	No	313 (43.9)
	Unknown	30 (4.2)
	Co‐infection with other BBV—n (%)	
	HIV and or HBV	8 (1.0)
	None	705 (99.0)

	Treated via pragmatic trial—n (%)	
	Yes	263 (36.9)
	No	450 (63.1)
	Receiving OST prior to treatment—n (%)	
	Yes	551 (77.3)
No	162 (22.7)

Abbreviations: BBV, blood‐borne virus; HBV, hepatitis B virus; HCV, hepatitis C virus; HIV, human immunodeficiency virus; IQR, interquartile range; OST, opioid substitution therapy; Peg‐IFN, pegylated interferon; people who inject drugs; PWID; SIMD, Scottish Index of Multiple Deprivation.

^a^ Demographic data n = 662.

^b^ Pre‐treatment data n = 713.

The majority were treatment naïve prior to the scaleup and, among those who were treatment experienced, 9 in 10 treatments were based on pegylated interferon (Peg‐IFN). Most (88.4%, Table 2) completed their course of DAAs. Figure 1 shows outcomes by treatment initiation, and cumulative treatment and post‐treatment outcomes.

**TABLE 2:** Intention‐to‐treat (n = 713) and per‐protocol (n = 630) sustained virologic response, treatment initiations, completions, and loss to follow‐up by treatment pathway, for people who inject drugs in Tayside 2017‐20

	Conventional care	Drug treatment centres^a^	Community pharmacies^a^	Needle exchanges^a^	Nurse‐led community clinics^a^	Prisons^a^	Total
Intention‐to‐treat							
Treatments initiated—n (%)	91 (12.8)	46 (6.4)	144 (20.2)	205 (28.8)	124 (17.4)	103 (14.4)	713 (100)
Treatments complete—n (%)	83 (91.2)	41 (89.1)	140 (97.2)	160 (78.0)	112 (90.3)	94 (91.3)	630 (88.4)
SVR—n (%)	74 (81.3)	34 (73.9)	131 (91.0)	155 (75.6)	102 (82.3)	81 (78.6)	577 (80.9)
No SVR—n (%)	17 (18.7)	12 (26.1)	13 (9.0)	50 (24.4)	22 (17.7)	22 (21.4)	140 (19.1)
SVR exc. Unknown^b^—%	91.4	87.2	95.6	85.6	96.2	93.1	91.4
Loss to follow‐up^c^—n (%)	10 (12.5)	7 (8.8)	7 (8.8)	22 (27.5)	17 (21.2)	17 (21.2)	80 (100)
Per‐protocol^d^							
SVR—n (%)	70 (84.3)	32 (78.0)	129 (92.1)	133 (83.1)	96 (85.7)	76 (80.9)	536 (85.1)
SVR exc. Unknown^e^—%	95.9	94.1	96.3	94.3	98.0	95.0	95.7
No SVR—n (%)	13 (15.7)	9 (22.0)	11 (7.9)	27 (16.9)	16 (14.3)	18 (19.1)	94 (14.9)
Loss to follow‐up^c^—n (%)	9 (14.5)	7 (11.3)	4 (6.4)	17 (27.4)	12 (19.4)	13 (21.0)	62 (100)

Abbreviations: exc., excluding; SVR, sustained virologic response.

^a^ Novel care pathway.

^b^ n = 631.

^c^ Those lost to follow‐up but believed to reside locally, and those who moved.

^d^ Those who completed treatment.

^e^ n = 560.

577 SVRs (80.9%) were obtained in the ITT population (n = 713). Eighty‐two cases did not have an SVR test (lost to follow‐up, deceased or moved). Excluding those missing an SVR test in the ITT group, SVR was 91.4% (Table 2). In the PP population (n = 630), 536 SVRs were obtained (85.1%). Excluding 62 PP cases that did not have an SVR test, this rose to 95.7%. Loss to follow up (LTFU) varied substantially across pathways (Table 2).

Of treatment completers, eight died prior to SVR testing. Of treatment non‐completers (n = 83), 41 (49.4%) obtained SVR, 30 (36.1%) did not and 12 (14.5%) were missing SVR data. Of those 12, 8 were LTFU; 3 moved and 1 died. For treatment non‐completers with documented SVR tests (n = 71), 57.7% obtained a cure.

### 3.1 HCV re‐infection and follow‐up RNA testing

During the study, 236 cases among 231 individuals were followed‐up for re‐infection. Of those who obtained SVR, 39% (227/577) had at least one follow‐up RNA test. Re‐infection occurred 39 times, 9 detected at SVR, over 256.57 PY of follow‐up, yielding a rate of 15.20 per 100 PY (95% CI 10.81‐20.78). Among those who reported recent injecting, there were 142 cases among 140 individuals over 175.43 PY, yielding a rate of 14.82 per 100 PY (95%CI 9.68‐21.72). Among re‐infections, 25 cases were re‐treated: 17 obtained SVR, four did not, and four were LTFU. Of re‐infected individuals, incidence was highest among those initially treated through needle exchanges (Table 3). Of the 25 re‐treated cases, 23 (92%) had completed the course of DAAs for their initial infection. Fourteen cases did not have further treatment within the study timeline; four of whom died, with the remaining 10 LTFU.

**TABLE 3:** Clinical pathways for HCV re‐infected PWID in Tayside 2017‐20

Clinical pathway	n (%)	PY	Incidence per 100 PY^a^
Pathway prior to HCV re‐infection (n = 39)			
Hospital	0 (0.0)	22.93	0.00 (0.00‐16.09)^b^
Community pharmacies	12 (30.8)	92.25	13.01 (6.72‐22.73)
Needle exchanges	19 (48.7)	64.08	29.65 (17.85‐46.30)
Other^c^	8 (20.5)	77.31	10.35 (4.47‐20.39)

Abbreviations: HCV, hepatitis c virus infection; PWID, people who inject drugs; PY, person‐years of observation.

^a^ 95% confidence interval indicated in brackets.

^b^ One‐sided *p*, 97.5% confidence interval.

^c^ For disclosure reasons, drug treatment centres; nurse‐led community clinics; and prison pathways are combined.

After obtaining SVR, 87 (15%) of 577 cases had repeat RNA testing in the first 12 months, in line with policy. Within two years of SVR, 110 (19%) of 577 had follow‐up testing. Of those, 20 (18%) reflected continued follow‐up from the previous year, in line with policy. Fifteen were tested within three years, and four within four years. The remainder either did not receive follow‐up testing or had non‐standard test intervals (eg <12 months from SVR).

### 3.2 Mortality

Fifty‐four treated individuals died over a period of 1,553.04 PY, yielding an all‐cause mortality rate of 3.48 per 100 PY (95% CI 2.61‐4.54); 29 were drug related, yielding an estimated drug‐related mortality rate of 1.87 per 100 PY (95% CI 1.25‐2.68). Four (9%) deaths were liver‐related, due to: hepatocellular carcinoma (2); liver metastases and accompanying causes (2). Forty‐four (81.5%) patients died following SVR, while 10 died before SVR sample could be obtained.

### 3.3 Treatment pathways

**FIGURE 2:**
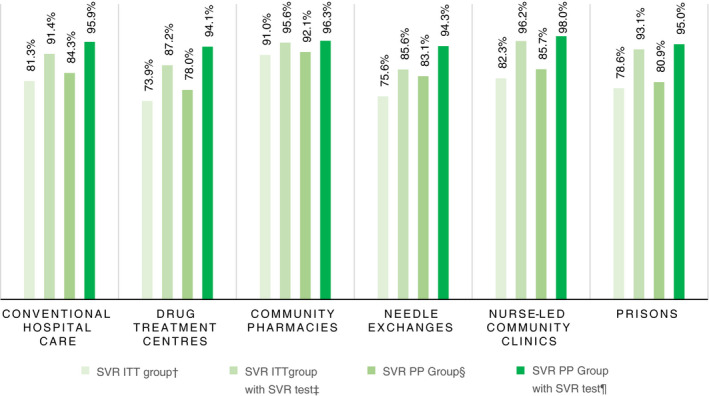
Proportion of sustained virologic response among people who inject drugs by treatment pathway. ^†^All treatment initiations, those without SVR test assumed treatment failures (n = 713). ^‡^All treatment initiations with a related SVR test (n = 631). ^§^All treatments were completed, those without SVR test assumed treatment failures (n = 630). ^¶^All treatments were confirmed completed, with a documented SVR test (n = 560). SVR, sustained virologic response; ITT, intention‐to‐treat; PP, per protocol

Needle exchanges initiated the highest proportion of treatments (Table 2). Collectively needle exchanges and community pharmacies initiated almost half of all treatments. Treatment completion was highest in pharmacies and lowest in needle exchanges, while LTFU was highest in needle exchanges and lowest in pharmacies. SVR seemed to vary across pathways in the ITT group. However, among treatment completers with a test, SVR rates stabilised in excess of 90% (Figure 2).

Results for pathway‐denominated logistic regressions are presented in Table 4, with full tabulation in Data S1 (Sections 2 and 3).

**TABLE 4:** Logistic regression modelling of sustained virologic response by treatment pathway

Model	N	Variable	SVR—n (%)	Unadjusted		Adjusted	
				OR (95% CI)	*P*	*a*OR (95% CI)	*P*
ITT	713	Treatment pathway [hospital]	74 (81.3)				
		Drug treatment centres	34 (73.9)	0.65 (0.28‐1.51)	0.318	0.60 (0.25‐1.42)	0.243
		Community pharmacies	131 (91.0)	2.32 (1.07‐5.03)	<0.05	2.16 (0.95‐4.90)	0.066
		Needle exchanges	155 (75.6)	0.71 (0.39‐1.32)	0.280	0.63 (0.32‐1.23)	0.172
		Nurse‐led community clinics	102 (82.3)	1.07 (0.53‐2.15)	0.860	0.97 (0.46‐2.02)	0.930
		Prison	81 (78.6)	0.85 (0.42‐1.72)	0.643	0.63 (0.29‐1.38)	0.247
ITT with SVR test	631	Treatment pathway [hospital]	74 (91.4)				
		Drug treatment centres	34 (87.2)	0.64 (0.19‐2.17)	0.477	0.61 (0.17‐5.16)	0.440
		Community pharmacies	131 (95.6)	2.07 (0.67‐6.38)	0.207	1.85 (0.56‐6.18)	0.315
		Needle exchanges	155 (85.6)	0.56 (0.23‐1.36)	0.202	0.50 (0.19‐1.33)	0.496
		Nurse‐led community clinics	102 (96.2)	2.41 (0.68‐8.54)	0.172	2.14 (0.58‐7.97)	0.256
		Prison	81 (93.1)	1.28 (0.41‐3.97)	0.673	1.10 (0.31‐3.88)	0.881
PP	630	Treatment pathway [hospital]	70 (84.3)				
		Drug treatment centres	32 (78.0)	0.66 (0.26‐1.70)	0.390	0.57 (0.21‐1.55)	0.274
		Community pharmacies	129 (92.1)	2.18 (0.93‐5.12)	0.074	1.93 (0.77‐4.87)	0.163
		Needle exchanges	133 (83.1)	0.92 (0.44‐1.88)	0.280	0.75 (0.34‐1.67)	0.480
		Nurse‐led community clinics	96 (85.7)	1.11 (0.50‐2.47)	0.789	0.96 (0.41‐2.24)	0.928
		Prison	76 (80.9)	0.78 (0.36‐1.72)	0.543	0.52 (0.22‐1.27)	0.152
PP with SVR test	560	Treatment pathway [hospital]	70 (95.9)				
		Drug treatment centres	32 (94.1)	0.69 (0.11‐4.31)	0.687	0.46 (0.07‐3.17)	0.429
		Community pharmacies	129 (96.3)	1.11 (0.26‐4.77)	0.893	0.62 (0.12‐3.18)	0.569
		Needle exchanges	133 (94.3)	0.71 (0.18‐2.77)	0.621	0.40 (0.09‐1.87)	0.246
		Nurse‐led community clinics	96 (98.0)	2.06 (0.34‐12.64)	0.436	1.34 (0.20‐8.88)	0.760
		Prison	76 (95.0)	0.71 (0.18‐3.78)	0.793	0.96 (0.71‐1.01)	0.107

Abbreviations: aOR, adjusted odds ratio; ITT, intention‐to‐treat; OR, odds ratio; PP, per protocol; SVR, sustained virologic response.

The conventional hospital pathway is the reference group for all models.

The unadjusted model in the ITT population (n = 713) implied that those treated through community pharmacies had higher odds of achieving SVR (OR 2.32 [1.07‐5.03], *P* ≤ 0.05). However, adjusted for patient characteristics, this became marginally non‐significant (OR 2.17 [.95‐4.94], *P* = 0.066). There were no significant differences between pathways in the ITT group who attended for SVR. In the PP groups with (n = 630) and without (n = 560) SVR test, there were also no significant differences in SVR between pathways.

In the PP group, adjusted for age and gender, the model implied that female gender may be associated with reduced odds of SVR (OR 0.59 [.35‐1.01], *P =* 0.056). Of females who did not obtain SVR in this model, for 66.7% (18/27) this was due to LTFU after treatment completion. Suggesting that, following treatment completion, follow‐up for females may require attention across all pathways, as the effect fell away in the PP group with an SVR test (Data S1, Tables 2.8 and 2.9). Models combining all community pathways into one comparator were non‐significant across all groups (Data S1, Section 3).

**FIGURE 3:**
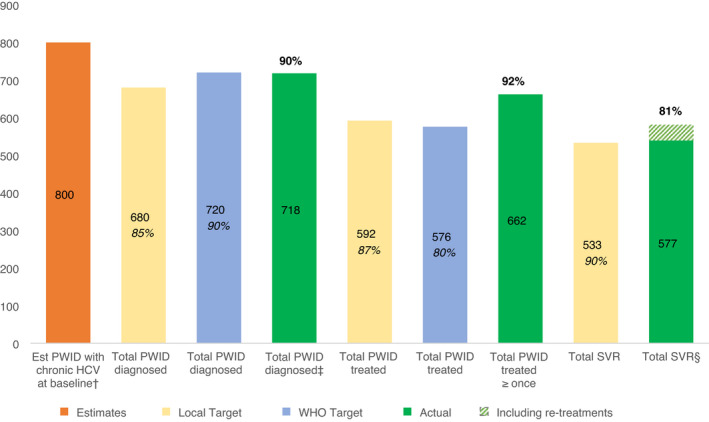
Estimated HCV Care Cascade in Tayside population of People Who Inject Drugs, alongside targets and outcomes for HCV diagnosis and treatment in the scaleup period. Est, estimated; HCV, hepatitis c virus; PWID, people who inject drugs; SVR, sustained virologic response; WHO, World Health Organization. Note: percentages rounded to the nearest whole number. Intention‐to‐treat and per‐protocol rates were reported for SVR. ^†^Based on 29% estimated chronic infection in a population of 2,800 people who inject drugs. ^‡^Total with ≥one HCV RNA positive test from June 2016 to April 2020 on the national clinical database. 2016 diagnoses included as they will have been eligible for treatment at beginning of treatment scaleup in 2017. ^§^Proportion of all treated cases (not treated individuals). SVR was 91% in cases with a documented test

Figure 2 shows the estimated HCV cascade of care in Tayside, measured against local and WHO targets. It suggests that Tayside diagnosed the majority of the estimated infected PWID population and met the targets set for regional scaleup. Further, WHO diagnosis and treatment targets appear to have been met in line with baseline infection estimates (Figure 3).

## 4 DISCUSSION

Rapid regional HCV treatment scale‐up in PWID was achieved in Tayside. There were over 700 treatments across six pathways in three years, a close to two‐fold increase on previously high levels of HCV treatment among PWID in Tayside. The estimated care cascade shows that 90% of the estimated HCV‐infected PWID population were diagnosed and 92% of those initiated treatment. SVR in the ITT group was over 80% and exceeded 90% in those with a documented test. Among treatment completers, SVR exceeded 85% and was over 95% for those with a documented test. Pharmacies and needle exchanges made substantial contributions, delivering almost half of all treatments. Our data adds strong population‐level evidence to the literature supporting HCV care in community pharmacies.^21^, ^23^, ^24^, ^25^

Our findings suggest a high level of attrition at the post‐treatment follow‐up point among our cohort of PWID. This contrasts with data from, for example, the Icelandic national scaleup among PWID, which reported minimal attrition at each step of the HCV cascade of care.^26^ The statistical analyses initially implied that receiving treatment through a community pharmacy was associated with higher odds of SVR. However, in the ITT population who attended for SVR, there were no significant differences between community pathways and hospital care. This implies differences in SVR were caused by variation in follow‐up and that, irrespective of treatment completion, pharmacies provided superior follow‐up for SVR testing. The models further suggested that SVR did not vary by treatment pathway or patient characteristics among those who completed treatment. One part‐adjusted model was significant which implied that when accounting for treatment pathway, age and gender, female gender was weakly associated with reduced odds of SVR. Consequently, targeted follow‐up initiatives may be required to improve follow‐up for SVR testing among females across all pathways.

The data indicate high deprivation, in line with national estimates, and reinforces evidence that HCV disproportionately impacts the most disadvantaged.^27^, ^28^, ^29^ This Tayside cohort is relatively young, with low incidence of BBV co‐morbidity. However, mortality risk in the population was notable, and over half of deaths were drug related. This aligns with reports suggesting Tayside has the second‐highest rate of DRDs in Scotland and implies that service improvements are required to reduce drug‐related harms.^30^, ^31^ Compared to a recent cohort study of drug‐related mortality among HCV‐infected PWID from 2009‐18—in which Tayside resident PWID had an equivalent drug‐related morality rate of 1.02 per 100 PY, the highest of the four4 largest Scottish health boards, and all Scottish PWID had an equivalent rate of 1.24 per 100 PY—our observed mortality rate is concerningly high.^32^ Ongoing re‐infection and mortality risks in our cohort suggest substance misuse and harm reduction services for PWID in Tayside must improve.

The high proportion of cure adds valuable real‐world evidence to existing literature demonstrating the efficacy of DAAs for treating HCV in PWID.^5^, ^6^ The highest proportion of SVR (excluding unknowns) in ITT analysis was in the nurse‐led community pathway. In this pathway, diagnosis and DAA prescribing are undertaken by nurses. Similarly, in the pharmacy pathway, diagnosis and treatment can be wholly facilitated by the pharmacist through the use of simplified DAA prescribing algorithms and the use of dried blood spot sampling. The outcomes in our cohort strengthen the case for task‐shifting elements of care which can be completed in the community into the community for PWID.^33^, ^34^, ^35^

Our data augments the literature suggesting re‐treatment following re‐infection should be offered without stigma or discrimination.^36^, ^37^ Despite the high re‐infection rate among the subset with follow‐up—which aligns with national re‐infection estimates—recent data demonstrated a reduction in chronic HCV among PWID in Tayside, consistent with the rapid sale‐up.^38^, ^39^ The high re‐infection rate among needle exchange clients in this cohort is consistent with prior longitudinal studies from Tayside—which predominantly studied those treated with Peg‐IFN based therapies since 1998—but the overall rate observed in our study is considerably higher, and may imply a temporal change in the risk of re‐infection concurrent to the shift to DAA‐only treatment.^40^ This rate, particularly compared to a recent meta‐analysis which found lower re‐infection rates (6.2/100 PY; 95% CI 4.3‐9.0) among recent injectors, is not surprising in this early phase.^41^ It implies we treated those most likely to drive HCV transmission locally, and we hypothesise it will decrease as the population level benefits of the scaleup are realised and additional follow‐up is completed. Continued follow‐up and re‐treatment of re‐infection in coming years is the basis of the TasP study to drive chronic HCV to below 10%.

Data reported here affirms existing evidence that completing treatment with DAAs elicits high SVR rates among PWID.^5^ This suggests that if HCV treatment is adequately supported, SVR will likely be achieved. Recent treatment guidelines noted the potential to omit SVR testing.^36^ Our data suggest that, for those who complete treatment, it may be feasible to omit SVR testing whilst assuming treatment was successful. This may stimulate further discussion on whether the primary goal of HCV treatment is obtaining SVR or confirmed treatment completion. However, we acknowledge that omitting SVR testing may only be desirable in simpler cases of infection or in contexts where re‐infection is low.^42^

### 4.1 Limitations

This study is limited by its observational nature, its reliance on routinely collected data, and the lack of a comparator cohort, which is the basis of the next phase of the study. During data collection, some assumptions were made, for example if date of infection was unavailable a surrogate was used, such as treatment initiation date, which may have affected person‐time estimates. Further, most cases that resulted in SVR were missing post‐SVR RNA testing, which limited re‐infection estimates. Finally, the NHS is free for patients: possibly limiting international transferability of the care model. However, this study's strength lies in its rich description of the PWID population and the unique investigation of TasP in the context of HCV elimination on a regional level using real‐world data.

## 5 CONCLUSIONS

Our analyses indicate that Tayside achieved pre‐specified local diagnosis, treatment, and SVR targets set for the TasP scale‐up programme. Further, Tayside has met WHO testing and treatment targets having diagnosed 90% of the estimated PWID population with HCV, and treated over 80% of cases.^1^ Our data implies that rapid regional scale‐up of HCV treatment for PWID can be achieved through novel multi‐stakeholder pathways focussed on shifting testing, DAA prescribing, and treatment into community settings. To our knowledge, is the first real‐world study from the United Kingdom to demonstrate the effects of rapid regional HCV treatment scaleup among PWID, highlight the benefits of utilising non‐specialist prescribers regionally, and quantitatively compare novel community pathway contributions to scaling up HCV treatment.

The re‐infection rate, and proportion of DRDs, suggests we reached the highest‐risk individuals for transmission of HCV and implies that harm reduction and substance misuse services in the region need strengthening. Other areas targeting PWID to achieve elimination should provide substantial‐related care in these respects alongside comprehensive testing and easy access to treatment.

## AUTHORSHIP

*Guarantor of the article*: Christopher J Byrne.

*Author Contributions*: CJB co‐ordinated the study, collected data, performed statistical analyses and wrote the initial manuscript draft. LB co‐ordinated the study and collected data. SKI managed the study. ER advised on testing data. AR, DJG, MH, SH and JFD collaboratively conceived, designed, planned and executed the trial. All authors critically revised the manuscript for intellectual content and approved the final version.

## Supporting information

Supplementary Material

## Data Availability

The data underpinning this study were obtained from routinely updated NHS health records in line with approval granted by the NHS Caldicott Guardian. The individuals to whom the data pertains did not explicitly consent to its use for research purposes. Therefore, it is not possible for the authors to share this data. However, interested parties can make specific requests to NHS Tayside Information Governance by email on: informationgovernance.tayside@nhs.scot.
