# Supplementary material for: Real‐world outcomes of rapid regional hepatitis C virus treatment scale‐up among people who inject drugs in Tayside, Scotland
Source: Aliment Pharmacol Ther. 2021 Dec 8;55(5):568–79. doi: 10.1111/apt.16728 (PMC9300005; doi:10.1111/apt.16728)
Supplement: Supplementary file 1 — Supplementary Material [file APT-55-568-s001.docx]

**Section 1** – Descriptive Pre-treatment Characteristics

| **Table 1.1**: Descriptive Pre-treatment Characteristics broken down by pathway for intention-to-treat group (n=713) | | | | | | |
| --- | --- | --- | --- | --- | --- | --- |
|  | **Genotype** | | | | | |
|  | *Hospital* | *Drug Treatment* | *Pharmacies* | *Needle exchanges* | *Nurse-led clinics* | *Prison* |
| 1 | 37 | 18 | 47 | 84 | 46 | 48 |
| 3 | 51 | 26 | 97 | 115 | 75 | 39 |
| 2 or 4 | 2 | 2 | 0 | 5 | 2 | 3 |
| Unknown | 1 | 0 | 0 | 1 | 1 | 13 |
| **Liver Cirrhosis** | | | | | | |
|  | *Hospital* | *Drug Treatment* | *Pharmacies* | *Needle exchanges* | *Nurse-led clinics* | *Prison* |
| Yes | 24 | 1 | 6 | 13 | 17 | 4 |
| No | 67 | 45 | 138 | 192 | 107 | 97 |
| Unknown | 0 | 0 | 0 | 0 | 0 | 2 |
|  | **Report injecting in 12 months prior to treatment** | | | | | |
|  | *Hospital* | *Drug Treatment* | *Pharmacies* | *Needle exchanges* | *Nurse-led clinics* | *Prison* |
| Within last 12 months | 20 | 20 | 64 | 169 | 55 | 42 |
| Prior to last 12 months | 68 | 26 | 78 | 29 | 69 | 43 |
| Not known | 3 | 0 | 2 | 7 | 0 | 18 |
|  | **Co-infection with other BBV** | | | | | |
|  | *Hospital* | *Drug Treatment* | *Pharmacies* | *Needle exchanges* | *Nurse-led clinics* | *Prison* |
| Yes | 5 | 0 | 0 | 1 | 1 | 1 |
| No | 86 | 46 | 144 | 204 | 123 | 102 |
|  | **Clinical trial participation** | | | | | |
|  | *Hospital* | *Drug Treatment* | *Pharmacies* | *Needle exchanges* | *Nurse-led clinics* | *Prison* |
| Yes | 0 | 0 | 143 | 120 | 0 | 0 |
| No | 91 | 46 | 1 | 85 | 124 | 103 |
|  | **Opioid Substitution Therapy receipt** | | | | | |
|  | *Hospital* | *Drug Treatment* | *Pharmacies* | *Needle exchanges* | *Nurse-led clinics* | *Prison* |
| Yes | 43 | 43 | 144 | 159 | 97 | 65 |
| No | 48 | 3 | 0 | 46 | 27 | 38 |

| **Table 1.2**: Descriptive Pre-treatment Characteristics broken down by pathway for per-protocol group (n=630) | | | | | | |
| --- | --- | --- | --- | --- | --- | --- |
|  | **Genotype** | | | | | |
|  | *Hospital* | *Drug Treatment* | *Pharmacies* | *Needle exchanges* | *Nurse-led clinics* | *Prison* |
| 1 | 34 | 16 | 46 | 65 | 40 | 42 |
| 3 | 46 | 23 | 94 | 92 | 69 | 37 |
| 2 or 4 | 2 | 2 | 0 | 2 | 2 | 2 |
| Unknown | 1 | 0 | 0 | 1 | 1 | 13 |
| **Liver Cirrhosis** | | | | | | |
|  | *Hospital* | *Drug Treatment* | *Pharmacies* | *Needle exchanges* | *Nurse-led clinics* | *Prison* |
| Yes | 22 | 1 | 6 | 11 | 16 | 4 |
| No | 22 | 1 | 6 | 11 | 16 | 4 |
| Unknown | 0 | 0 | 0 | 0 | 0 | 2 |
|  | **Report injecting in 12 months prior to treatment** | | | | | |
|  | *Hospital* | *Drug Treatment* | *Pharmacies* | *Needle exchanges* | *Nurse-led clinics* | *Prison* |
| Within last 12 months | 18 | 19 | 63 | 129 | 47 | 38 |
| Prior to last 12 months | 62 | 22 | 75 | 26 | 65 | 39 |
| Not known | 3 | 0 | 2 | 5 | 0 | 17 |
|  | **Co-infection with other BBV** | | | | | |
|  | *Hospital* | *Drug Treatment* | *Pharmacies* | *Needle exchanges* | *Nurse-led clinics* | *Prison* |
| Yes | 5 | 0 | 0 | 1 | 1 | 1 |
| No | 78 | 41 | 140 | 159 | 111 | 93 |
|  | **Clinical trial participation** | | | | | |
|  | *Hospital* | *Drug Treatment* | *Pharmacies* | *Needle exchanges* | *Nurse-led clinics* | *Prison* |
| Yes | 0 | 0 | 139 | 87 | 0 | 0 |
| No | 83 | 41 | 1 | 73 | 112 | 94 |
|  | **Opioid Substitution Therapy receipt** | | | | | |
|  | *Hospital* | *Drug Treatment* | *Pharmacies* | *Needle exchanges* | *Nurse-led clinics* | *Prison* |
| Yes | 40 | 39 | 140 | 131 | 89 | 59 |
| No | 43 | 2 | 0 | 29 | 23 | 35 |

**Section 2** – full logistic regression tabulation.

Treatment pathway was the predictor of interest for all analyses. In adjusted analyses, age, sex, genotype, and cirrhosis status were included in a stepwise approach. Details of the structure of covariates are in Table 2.1.

| **Table 2.1**: Details of covariates for adjusted analyses. | | | |
| --- | --- | --- | --- |
| **Categorical variable** | **Structure**^a^ | | *df* |
| Treatment pathway | *Hospital care* | | 5 |
|  | Drug Treatment centres | |  |
|  | Community Pharmacies | |  |
|  | Needle exchanges | |  |
|  | Nurse-led community clinics | |  |
|  | Prison | |  |
| Age at treatment (n=713) | Mean (95% CI): | 40.35 (39.70 – 40.99) | n/a |
|  | Median: | 39.00 |  |
|  | Min–Max: | 21 – 80 |  |
| Age at treatment (n=630) | Mean (95% CI): | 40.27 (39.58 – 40.96) | n/a |
|  | Median: | 39.00 |  |
|  | Min–Max: | 21 – 80 |  |
| Age at treatment (n=631) | Mean (95% CI): | 40.72 (40.03 – 41.40) | n/a |
|  | Median: | 39.00 |  |
|  | Min–Max: | 21 – 80 |  |
| Age at treatment (n=560) | Mean (95% CI): | 40.64 (39.91 – 41.37) | n/a |
|  | Median: | 39.00 |  |
|  | Min–Max: | 21 – 80 |  |
| Sex | *Male* | | 1 |
|  | Female | |  |
| Genotype | *1* | | 2 |
|  | 2, 4, Unknown | |  |
|  | 3 | |  |
| Cirrhosis | *Cirrhosis* | | 1 |
|  | No cirrhosis | |  |
| ^a^Reference categories are italicised.  **Abbreviations:** df, degrees of freedom; Min, minimum; Max, maximum. | | | |

**Analysis 1**

An unadjusted logistic regression was performed to ascertain the effects of receiving DAA treatment in community pathways compared to the standard hospital pathway on odds of achieving SVR in the intention-to-treat (ITT) group (n=713). This group included treatment completers and non-completers and those with or without an SVR test. Those without an SVR test were assumed treatment failures. The model was statistically significant, *χ*^2^(5) = 16.58, *p* = .005, and explained 37% (Nagelkerke R^2^) of the variance in SVR. 80.9% of cases were correctly classified. Individuals treated through the community pharmacy pathway had increased odds of achieving SVR compared to standard care (Table 2.2).

| **Table 2.2**: Results of unadjusted logistic regression in the ITT group, missing SVR assumed failed (n=713). | | | | | | |
| --- | --- | --- | --- | --- | --- | --- |
| Variable | SE | *X^2^* | SVR – n (%) | OR | 95% CI | *p* |
| Treatment pathway [hospital] |  | 14.22 | 74 (81.3) |  |  |  |
| *Drug treatment centres* | .430 | .996 | 34 (73.9) | .65 | .28 – 1.51 | .318 |
| *Community pharmacies* | .396 | 4.49 | 131 (91.0) | 2.32 | 1.07 – 5.03 | <.05 |
| *Needle exchanges* | .314 | 1.12 | 155 (75.6) | .71 | .39 – 1.32 | .280 |
| *Nurse-led clinics* | .357 | .031 | 102 (82.3) | 1.07 | .53 – 2.15 | .860 |
| *Prison* | .361 | .215 | 81 (78.6) | .85 | .42 – 1.72 | .643 |
| Constant | .239 | 29.91 |  |  |  | <.001 |
| **Abbreviations**: ITT, intention-to-treat; SE, standard error; *X^2^*, Wald statistic; SVR, sustained virologic response; OR, odds ratio. | | | | | | |

**Analysis 2**

The logistic regression model was adjusted in a stepwise manner in the same ITT group (Table 2.3). Age was assessed for linearity to its log odds and found to be satisfactory.

At step 1, the model was statistically significant, *χ*^2^(6) = 17.67, *p* = .007, and explained 39% (Nagelkerke R^2^) of the variance in SVR. The odds ratio for the community pharmacy pathway decreased by 12% compared to unadjusted analyses and it became marginally non-significant due to the inclusion of age, which itself was not significant, as a covariate.

At step 2, the model retained overall statistical significance, *χ*^2^(7) = 19.69 *p* = .006, and explained 44% (Nagelkerke R^2^) of the variance in SVR. The *Community Pharmacy* indicator trended toward significance with the inclusion of sex, which itself was not significant. The model was a slightly improved fit to the data.

At step 3, the overall statistical significance of the model was *χ*^2^(9) = 20.63 *p* = .014, and explained 46% (Nagelkerke R^2^) of the variance in SVR. The *Community Pharmacy* indicator trended further toward significance with the inclusion of Genotype, which itself was not significant. The model was not an improved fit to the data.

At step 4, the model retained statistical significance, *χ*^2^(10) = 20.63 *p* = .024. The model was not an improved fit to the data, with explained variance of SVR remaining at 46% (Nagelkerke R^2^). There were no substantial changes in effects among covariates due to inclusion of cirrhosis status, which itself was not statistically significant. Case classification rate (80.9% correct) was unchanged in any step of the adjusted analyses compared to unadjusted analysis.

The fully adjusted model implies that in the ITT population, even when accounting for age, sex, genotype, and cirrhosis status, being treated through community pharmacies *may* lead to increased odds of obtaining SVR.

| **Table 2.3**: Results of adjusted logistic regressions in the ITT group, missing SVR assumed failed (n=713). | | | | | | | |
| --- | --- | --- | --- | --- | --- | --- | --- |
| Step | Variable | SE | *X^2^* | SVR – n (%) | aOR | 95% CI | *p* |
| 1 | Treatment pathway [hospital] |  | 14.712 | 74 (81.3) |  |  |  |
|  | *Drug treatment centres* | .442 | 1.44 | 34 (73.9) | .59 | .25 – 1.40 | .231 |
|  | *Community pharmacies* | .415 | 2.96 | 131 (91.0) | 2.04 | .91 – 4.60 | .085 |
|  | *Needle exchanges* | .342 | 1.95 | 155 (75.6) | .62 | .32 – 1.21 | .163 |
|  | *Nurse-led clinics* | .375 | .02 | 102 (82.3) | .95 | .46 – 1.98 | .886 |
|  | *Prison* | .386 | .64 | 81 (78.6) | .74 | .35 – 1.57 | .425 |
|  | Age at treatment | .012 | 1.10 |  | .99 | .97 – 1.01 | .295 |
|  | Constant | .650 | 10.29 |  | 8.05 |  | <.01 |
| 2 | Treatment pathway [hospital] |  | 15.55 | 74 (81.3) |  |  |  |
|  | *Drug treatment centres* | .443 | 1.35 | 34 (73.9) | .60 | .25 – 1.42 | .245 |
|  | *Community pharmacies* | .417 | 3.22 | 131 (91.0) | 2.11 | .93 – 4.78 | .073 |
|  | *Needle exchanges* | .344 | 1.88 | 155 (75.6) | .63 | .32 – 1.23 | .171 |
|  | *Nurse-led clinics* | .376 | .01 | 102 (82.3) | .96 | .46 – 2.01 | .960 |
|  | *Prison* | .393 | 1.07 | 81 (78.6) | .67 | .31 - .1.44 | .301 |
|  | Age at treatment | .012 | 1.68 |  | .98 | .96 – 1.01 | .195 |
|  | Sex [male] |  |  | 431 (81.6) |  |  |  |
|  | *Female* | .230 | 2.06 | 146 (78.9) | .72 | .46 – 1.13 | .152 |
|  | Constant | .672 | 11.82 |  | 10.08 |  | <.01 |
| 3 | Treatment pathway [hospital] |  | 16.22 | 74 (81.3) |  |  |  |
|  | *Drug treatment centres* | .444 | 1.36 | 34 (73.9) | .60 | .25 – 1.42 | .243 |
|  | *Community pharmacies* | .418 | 3.41 | 131 (91.0) | 2.16 | .95 – 4.90 | .065 |
|  | *Needle exchanges* | .344 | 1.87 | 155 (75.6) | .63 | .32 – 1.23 | .172 |
|  | *Nurse-led clinics* | .376 | .01 | 102 (82.3) | .97 | .46 – 2.02 | .930 |
|  | *Prison* | .397 | 1.34 | 81 (78.6) | .63 | .29 – 1.38 | .247 |
|  | Age at treatment | .012 | 1.64 |  | .72 | .46 – 1.13 | .155 |
|  | Sex [male] |  |  | 431 (81.6) |  |  |  |
|  | *Female* | .231 | 2.02 | 146 (78.9) | .72 | .46 – 1.13 | .155 |
|  | Genotype [1] |  |  | 228 (81.4) |  |  |  |
|  | *2,4,unknown* | .523 | .33 | 25 (83.3) | 1.35 | .49 – 3.77 | .564 |
|  | *3* | .203 | .38 | 324 (80.4) | .88 | .59 – 1.31 | .536 |
|  | Constant | .680 | 12.11 |  | 10.66 |  | .001 |
| 4 | Treatment pathway [hospital] |  | 16.21 | 74 (81.3) |  |  |  |
|  | *Drug treatment centres* | .447 | 1.32 | 34 (73.9) | .60 | .25 – 1.44 | .251 |
|  | *Community pharmacies* | .420 | 3.39 | 131 (91.0) | 2.17 | .95 – 4.94 | .066 |
|  | *Needle exchanges* | .346 | 1.83 | 155 (75.6) | .63 | .32 – 1.23 | .176 |
|  | *Nurse-led clinics* | .376 | .01 | 102 (82.3) | .97 | .43 – 2.03 | .932 |
|  | *Prison* | .400 | 1.31 | 81 (78.6) | .63 | .29 – 1.39 | .252 |
|  | Age at treatment | .013 | 1.57 |  | .72 | .46 – 1.13 | .156 |
|  | Sex [male] |  |  | 431 (81.6) |  |  |  |
|  | *Female* | .231 | 2.02 | 146 (78.9) | .72 | .46 – 1.13 | .156 |
|  | Genotype [1] |  | .91 | 228 (81.4) |  |  |  |
|  | *2,4,unknown* | .523 | .33 | 25 (83.3) | 1.35 | .49 – 3.77 | .564 |
|  | *3* | .203 | .38 | 324 (80.4) | .88 | .59 – 1.31 | .536 |
|  | Cirrhosis [yes] |  |  | 52 (80.0) |  |  |  |
|  | *No* | .354 | .00 | 525 (81.0) | .98 | .49 – 1.95 | .947 |
|  | Constant | .801 | 8.93 |  | 10.97 |  | <.005 |
| **Abbreviations**: ITT, intention-to-treat; SE, standard error; *X^2^*, Wald statistic; SVR, sustained virologic response; aOR, adjusted odds ratio. | | | | | | | |

**Analysis 3**

An unadjusted logistic regression was performed to ascertain the effects of receiving DAA treatment in community pathways as compared to the standard hospital pathway on odds of achieving SVR in the ITT group who received an SVR test (n=631), i.e. both treatment completers and non-completers were included, but those without an SVR test were excluded. The model was statistically significant, *χ*^2^(5) = 15.24, *p* = .009, and explained 54% (Nagelkerke R^2^) of the variance in SVR. 91.4% of cases were correctly classified. Individuals treated through the community pathways did not have increased odds of achieving SVR compared to standard care (Table 2.4).

| **Table 2.4**: Results of unadjusted logistic regression in the ITT group with an SVR test (n=631). | | | | | | |
| --- | --- | --- | --- | --- | --- | --- |
| Variable | SE | *X^2^* | SVR – n (%) | OR | 95% CI | *p* |
| Treatment pathway [hospital] |  | 13.80 | 74 (91.4) |  |  |  |
| *Drug treatment centres* | .621 | .51 | 34 (87.2) | .64 | .19 – 2.17 | .477 |
| *Community pharmacies* | .575 | 1.59 | 131 (95.6) | 2.07 | .67 – 6.38 | .207 |
| *Needle exchanges* | .449 | 1.63 | 155 (85.6) | .56 | .23 – 1.36 | .202 |
| *Nurse-led clinics* | .645 | 1.86 | 102 (96.2) | 2.41 | .68 – 8.54 | .172 |
| *Prison* | .579 | .18 | 81 (93.1) | 1.28 | .41 – 3.97 | .673 |
| Constant | .395 | 35.56 |  | 10.57 |  | <.001 |
| **Abbreviations**: ITT, intention-to-treat; SE, standard error; *X^2^*, Wald statistic; SVR, sustained virologic response; OR, odds ratio. | | | | | | |

**Analysis 4**

The regression model was adjusted in a stepwise manner in the same ITT group with an SVR test (Table 2.5). Age was assessed for linearity to its log odds and found to be satisfactory.

At step 1, the model was statistically significant, *χ*^2^(6) = 15.80, *p* = .015, and explained 56% (Nagelkerke R^2^) of the variance in SVR. There were no significant changes to the effects of community pathways and Age at Treatment was not statistically significant itself.

At step 2, the model retained overall statistical significance, *χ*^2^(7) = 16.14 *p* = .024, and explained 57% (Nagelkerke R^2^) of the variance in SVR. There were no substantial changes in effects among covariates due to inclusion of sex, which itself was not statistically significant.

At step 3, the overall model became marginally non-significant, *χ*^2^(9) = 16.42 *p* = .059, and explained 58% (Nagelkerke R^2^) of the variance in SVR. There were no substantial changes in effects among covariates due to inclusion of genotype, which itself was not statistically significant.

At step 4, the model continued to be statistically non-significant, *χ*^2^(10) = 16.61 *p* = .083, and explained 59% (Nagelkerke R^2^) of the variance in SVR. There were no substantial changes in effects among covariates due to inclusion of cirrhosis status, which itself was not statistically significant.

Case classification rate (91.4% correct) was unchanged in any step of the adjusted analyses compared to unadjusted analysis.

The fully adjusted model has similar implications to the unadjusted analysis: when accounting for age and sex, among those who were follow-up for an SVR test, there was no statistically significant difference in odds of achieving SVR between community pathways and hospital care. However, there is uncertainty around the additional effects of genotype and cirrhosis status as the models at steps 3 and 4 where non-significant (albeit marginally).

| **Table 2.5**: Results of adjusted logistic regression in the ITT group with an SVR test (n=631). | | | | | | | |
| --- | --- | --- | --- | --- | --- | --- | --- |
| Step | Variable | SE | *X^2^* | SVR – n (%) | *a*OR | 95% CI | *p* |
| 1 | Treatment pathway [hospital] |  | 14.17 | 74 (91.4) |  |  |  |
|  | *Drug treatment centres* | .640 | .75 | 34 (87.2) | .57 | .16 – 2.01 | .386 |
|  | *Community pharmacies* | .607 | .92 | 131 (95.6) | 1.79 | .55 – 5.89 | .337 |
|  | *Needle exchanges* | .497 | 2.16 | 155 (85.6) | .48 | .18 – 1.28 | .142 |
|  | *Nurse-led clinics* | .67 | 1.25 | 102 (96.2) | 2.11 | .57 – 7.84 | .263 |
|  | *Prison* | .619 | .02 | 81 (93.1) | 1.09 | .32 – 3.66 | .894 |
|  | Age at treatment | .018 | .57 |  | .99 | .95 – 1.02 | .451 |
|  | Constant | .986 | 9.46 |  | 20.73 |  | <.005 |
| 2 | Treatment pathway [hospital] |  | 14.14 | 74 (91.4) |  |  |  |
|  | *Drug treatment centres* | .642 | .71 | 34 (87.2) | .58 | .17 – 2.05 | .398 |
|  | *Community pharmacies* | .609 | .98 | 131 (95.6) | 1.83 | .44 – 6.03 | .322 |
|  | *Needle exchanges* | .499 | 2.16 | 155 (85.6) | .48 | .18 – 1.28 | .142 |
|  | *Nurse-led clinics* | .669 | 1.25 | 102 (96.2) | 2.16 | .57 – 7.86 | .263 |
|  | *Prison* | .630 | .00 | 81 (93.1) | 1.02 | .30 – 3.50 | .976 |
|  | Age at treatment | .018 | .75 |  | .98 | .95 – 1.02 | .387 |
|  | Sex [male] |  |  | 431 (91.7) |  |  |  |
|  | *Female* | .344 | .35 | 146 (90.7) | .82 | .42 – 1.60 | .556 |
|  | Constant | 1.02 | 9.68 |  | 24.18 |  | <.005 |
| 3 | Treatment pathway [hospital] |  | 14.02 | 74 (91.4) |  |  |  |
|  | *Drug treatment centres* | .642 | .71 | 34 (87.2) | .58 | .17 – 2.05 | .339 |
|  | *Community pharmacies* | .610 | .92 | 131 (95.6) | 1.79 | .54 – 5.93 | .339 |
|  | *Needle exchanges* | .499 | 2.14 | 155 (85.6) | .48 | .18 – 1.28 | .144 |
|  | *Nurse-led clinics* | .670 | 1.25 | 102 (96.2) | 2.11 | .57 – 7.56 | .263 |
|  | *Prison* | .639 | .01 | 81 (93.1) | 1.07 | .31 – 3.73 | .920 |
|  | Age at treatment | .018 | .74 |  | .98 | .95 – 1.02 | .388 |
|  | Sex [male] |  |  | 431 (91.7) |  |  |  |
|  | *Female* | .344 | .35 | 146 (90.7) | .82 | .42 – 1.60 | .556 |
|  | Genotype [1] |  | .28 | 228 (90.8) |  |  |  |
|  | *2,4,unknown* | .669 | .06 | 25 (89.3) | .85 | .23 – 3.17 | .813 |
|  | *3* | .300 | .17 | 324 (92.0) | 1.13 | .63 – 2.04 | .683 |
|  | Constant | 1.03 | 9.14 |  | 22.67 |  | <.005 |
| 4 | Treatment pathway [hospital] |  | 13.85 | 74 (91.4) |  |  |  |
|  | *Drug treatment centres* | .648 | .60 | 34 (87.2) | .61 | .17 – 2.16 | .440 |
|  | *Community pharmacies* | .614 | 1.01 | 131 (95.6) | 1.85 | .56 – 6.18 | .315 |
|  | *Needle exchanges* | .503 | 1.95 | 155 (85.6) | .50 | .19 – 1.33 | .496 |
|  | *Nurse-led clinics* | .671 | 1.29 | 102 (96.2) | 2.14 | .58 – 7.97 | .256 |
|  | *Prison* | .643 | .02 | 81 (93.1) | 1.10 | .31 – 3.88 | .881 |
|  | Age at treatment | .019 | .89 |  | .98 | .947 – 1.02 | 345 |
|  | Sex [male] |  |  | 431 (91.7) |  |  |  |
|  | *Female* | .344 | .34 | 146 (90.7) | .82 | .42 – 1.61 | .819 |
|  | Genotype [1] |  | .27 | 228 (90.8) |  |  |  |
|  | *2,4,unknown* | .670 | .05 | 25 (89.3) | .87 | .23 – 3.22 | .828 |
|  | *3* | .300 | .17 | 324 (92.0) | 1.13 | .63 – 2.04 | .680 |
|  | Cirrhosis [yes] |  |  | 52 (92.9) |  |  |  |
|  | *No* | .581 | .18 | 525 (91.3) | .78 | .25 – 2.44 | .669 |
|  | Constant | 1.22 | 7.80 |  | 29.95 |  | .005 |
| **Abbreviations**: ITT, intention-to-treat; SE, standard error; *X^2^*, Wald statistic; SVR, sustained virologic response; aOR, adjusted odds ratio. | | | | | | | |

**Analysis 5**

An unadjusted logistic regression was performed in the per-protocol (PP) group (n=630). This group included treatment completers with or without an SVR test and assumed those without an SVR test were treatment failures. The model approached statistical significance, *χ*^2^(5) = 9.67, *p* = .085, and explained 27% (Nagelkerke R^2^) of the variance in SVR. 85.1% of cases were correctly classified. The community pharmacy pathway was the only one to approach statistical significance, although the overall *Treatment Pathway* variable was not significant (Table 2.6).

| **Table 2.6**: Results of unadjusted logistic regression in the PP group, missing SVR assumed failed (n=630). | | | | | | |
| --- | --- | --- | --- | --- | --- | --- |
| Variable | SE | *X^2^* | SVR – n (%) | OR | 95% CI | *p* |
| Treatment pathway [hospital] |  | 8.54 | 70 (84.3) |  |  |  |
| *Drug treatment centres* | .483 | .74 | 32 (78.0) | .66 | .26 – 1.70 | .390 |
| *Community pharmacies* | .436 | 3.19 | 129 (92.1) | 2.18 | .92 – 5.12 | .074 |
| *Needle exchanges* | .368 | .06 | 133 (83.1) | .92 | .44 – 1.88 | .280 |
| *Nurse-led clinics* | .405 | .07 | 96 (85.7) | 1.11 | .50 – 2.47 | .789 |
| *Prison* | .400 | .37 | 76 (80.9) | .78 | .36 – 1.72 | .543 |
| Constant | .302 | 31.08 |  | 5.39 |  | <.001 |
| **Abbreviations**: PP, per protocol; SE, standard error; *X^2^*, Wald statistic; SVR, sustained virologic response; OR, odds ratio. | | | | | | |

**Analysis 6**

The logistic regression model was adjusted in a stepwise approach for the same PP group (Table 2.7). Age was assessed for linearity to its log odds and found to be satisfactory.

At step 1, the model was remained statistically non-significant, *χ*^2^(6) = 11.29, *p* = .080, and explained 31% (Nagelkerke R^2^) of the variance in SVR. The community pharmacy pathway trended away from statistical significance and saw an 18% swing in odds ratio compared to the unadjusted analysis when controlled for *Age at Treatment*, which itself was not significant.

At step 2, the model became statistically significant, *χ*^2^(7) = 14.84, *p* = .038, and explained an increased 41% (Nagelkerke R^2^) of the variance in SVR. There were no substantial adjustments in effects of covariates at this step. However, sex was close to significant, implying that female gender was a negative predictor of SVR.

At step 3, the model reverted to non-significant, *χ*^2^(9) = 15.22, *p* = .085, with no meaningful changed in explained variance of SVR at (42%, Nagelkerke R^2^). There were no substantial changes in effects of covariates at this step and the addition of genotype was not significant.

At step 4, the model became remained non-significant, *χ*^2^(10) = 15.22, *p* = .124, and there was no change in explained variance in SVR. There were no substantial changes in effects of covariates at this step and the addition of cirrhosis status was not significant. Case classification rate (85.1% correct) was unchanged in any step of the adjusted analyses compared to unadjusted analysis.

The models imply that when assuming those with no SVR test were treatment failures among the cohort who completed treatment, controlled for age and gender, there is no statistically significant difference in SVR among community pathways compared to standard care. Further, it is implied female gender *may* reduce the odds of achieving SVR. However, the potential effects of genotype and cirrhosis status (steps 3 and 4) remain unclear as the models were not significant overall.

| **Table 2.7**: Results of adjusted logistic regressions in the PP group, missing SVR assumed failed (n = 630). | | | | | | | |
| --- | --- | --- | --- | --- | --- | --- | --- |
| Step | Variable | SE | *X^2^* | SVR – n (%) | aOR | 95% CI | *p* |
| 1 | Treatment pathway [hospital] |  | 8.75 | 70 (84.3) |  |  |  |
|  | *Drug treatment centres* | .500 | 1.30 | 32 (78.0) | .57 | .21 – 1.51 | .255 |
|  | *Community pharmacies* | .463 | 1.58 | 129 (92.1) | 1.79 | .72 – 4.44 | .209 |
|  | *Needle exchanges* | .402 | .52 | 133 (83.1) | .75 | .34 – 1.65 | .471 |
|  | *Nurse-led clinics* | .428 | .02 | 96 (85.7) | .94 | .41 – 2.17 | .879 |
|  | *Prison* | .436 | 1.10 | 76 (80.9) | .63 | .27 – 1.49 | .294 |
|  | Age at treatment | .014 | 1.63 |  | .98 | .96 – 1.01 | .202 |
|  | Constant | .772 | 11.19 |  | 13.21 |  | .001 |
| 2 | Treatment pathway [hospital] |  | 10.62 | 70 (84.3) |  |  |  |
|  | *Drug treatment centres* | .503 | 1.19 | 32 (78.0) | .58 | .22 – 1.55 | .275 |
|  | *Community pharmacies* | .468 | 1.90 | 129 (92.1) | 1.91 | .76 – 4.77 | .168 |
|  | *Needle exchanges* | .406 | .51 | 133 (83.1) | .75 | .34 – 1.66 | .168 |
|  | *Nurse-led clinics* | .431 | .01 | 96 (85.7) | .96 | .41 – 2.23 | .918 |
|  | *Prison* | .447 | 1.90 | 76 (80.9) | .54 | .23 – 1.30 | .169 |
|  | Age at treatment | .014 | 2.51 |  | .98 | .95 – 1.01 | .113 |
|  | Sex [male] |  |  | 408 (85.9) |  |  |  |
|  | *Female* | .272 | 3.66 | 128 (82.6) | .59 | .35 – 1.01 | .056 |
|  | Constant | .799 | 13.35 |  | 18.50 |  | <.001 |
| 3 | Treatment pathway [hospital] |  | 10.96 | 70 (84.3) |  |  |  |
|  | *Drug treatment centres* | .503 | 1.20 | 32 (78.0) | .58 | .22 – 1.55 | .273 |
|  | *Community pharmacies* | .469 | 1.99 | 129 (92.1) | 1.94 | .78 – 4.86 | .158 |
|  | *Needle exchanges* | .406 | .50 | 133 (83.1) | .75 | .34 – 1.67 | .482 |
|  | *Nurse-led clinics* | .431 | .01 | 96 (85.7) | .96 | .41 – 2.42 | .930 |
|  | *Prison* | .451 | 2.06 | 76 (80.9) | .52 | .22 – 1.27 | .151 |
|  | Age at treatment | .014 | 2.48 |  | .98 | .95 – 1.01 | .116 |
|  | Sex [male] |  |  | 408 (85.9) |  |  |  |
|  | *Female* | .273 | 3.53 | 128 (82.6) | .60 | .35 – 1.02 | .060 |
|  | Genotype [1] |  | .37 | 208 (85.6) |  |  |  |
|  | *2,4,unknown* | .587 | .07 | 22 (84.6) | 1.17 | .37 – 3.69 | .792 |
|  | *3* | .239 | .23 | 306 (84.8) | .89 | .60 – 1.43 | .635 |
|  | Constant | .810 | 13.48 |  | 19.53 |  | <.001 |
| 4 | Treatment pathway [hospital] |  | 10.96 | 70 (84.3) |  |  |  |
|  | *Drug treatment centres* | .508 | 1.20 | 32 (78.0) | .57 | .21 – 1.55 | .274 |
|  | *Community pharmacies* | .472 | 1.95 | 129 (92.1) | 1.93 | .77 – 4.87 | .163 |
|  | *Needle exchanges* | .408 | .50 | 133 (83.1) | .75 | .34 – 1.67 | .480 |
|  | *Nurse-led clinics* | .461 | .01 | 96 (85.7) | .96 | .41 – 2.24 | .928 |
|  | *Prison* | .454 | 2.05 | 76 (80.9) | .52 | .22 – 1.27 | .152 |
|  | Age at treatment | .015 | 2.29 |  | .98 | .95 – 1.01 | .130 |
|  | Sex [male] |  |  | 408 (85.9) |  |  |  |
|  | *Female* | .273 | 3.53 | 128 (82.6) | .60 | .35 – 1.02 | .060 |
|  | Genotype [1] |  | .37 | 208 (85.6) |  |  |  |
|  | *2,4,unknown* | .587 | .07 | 22 (84.6) | 1.17 | .37 – 3.69 | .793 |
|  | *3* | .239 | .23 | 306 (84.8) | .89 | .60 – 1.43 | .635 |
|  | Cirrhosis [yes] |  |  | 50 (83.3) |  |  |  |
|  | *No* | .395 | .00 | 486 (85.3) | 1.03 | .47 – 2.23 | .948 |
|  | Constant | .932 | 9.97 |  | 18.96 |  | <.05 |
| **Abbreviations**: ITT, intention-to-treat; SE, standard error; *X^2^*, Wald statistic; SVR, sustained virologic response; aOR, adjusted odds ratio. | | | | | | | |

**Analysis 7**

An unadjusted logistic regression was performed in the per-protocol (PP) group who received an SVR test (n=560). The model was not statistically significant, *χ*^2^(5) = 2.48, *p* = .779, and explained 15% (Nagelkerke R^2^) of the variance in SVR. 95.7% of cases were correctly classified. No community pathways were significant, and the overall *Treatment Pathway* variable was not significant (Table 2.8).

| **Table 2.8**: Results of unadjusted logistic regression in the PP group with an SVR test (n=560). | | | | | | |
| --- | --- | --- | --- | --- | --- | --- |
| Variable | SE | *X^2^* | SVR – n (%) | OR | 95% CI | *p* |
| Treatment pathway [hospital] |  | 2.16 | 70 (95.9) |  |  |  |
| *Drug treatment centres* | .937 | .16 | 32 (94.1) | .69 | .11 – 4.31 | .687 |
| *Community pharmacies* | .745 | .02 | 129 (96.3) | 1.11 | .26 – 4.77 | .893 |
| *Needle exchanges* | .693 | .24 | 133 (94.3) | .71 | .18 – 2.77 | .625 |
| *Nurse-led clinics* | .923 | .61 | 96 (98.0) | 2.06 | .34 – 12.64 | .436 |
| *Prison* | .782 | .07 | 76 (95.0) | .71 | .18 – 3.78 | .793 |
| Constant | .590 | 28.54 |  | 23.33 |  | <.001 |
| **Abbreviations**: PP, per protocol; SE, standard error; *X^2^*, Wald statistic; SVR, sustained virologic response; OR, odds ratio. | | | | | | |

**Analysis 8**

The logistic regression model was adjusted in a stepwise approach for the same PP group (Table 2.9). Age was assessed for linearity to its log odds and found to be satisfactory. At step 1, the model remained non-significant, *χ*^2^(6) = 5.15, *p* = .525, and explained 31% (Nagelkerke R^2^) of the variance in SVR. At step 2, the model remained non-significant, *χ*^2^(7) = 5.48, *p* = .601, and explained 33% (Nagelkerke R^2^) of the variance in SVR. At step 3, the model remained non-significant, *χ*^2^(9) = 6.83, *p* = .655, and explained 41% (Nagelkerke R^2^) of the variance in SVR. At step 4, the model remained non-significant, *χ*^2^(10) = 6.95, *p* = .730, and explained 41% (Nagelkerke R^2^) of the variance in SVR.

The results of these models suggest that in those who completed treatment and received an SVR test, there is no variability in SVR between pathways compared to the standard hospital pathway, even when adjusted for Age at Treatment, Sex, Genotype, and cirrhosis status.

| **Table 2.9**: Results of adjusted logistic regression in the PP group with an SVR test (n=560). | | | | | | | |
| --- | --- | --- | --- | --- | --- | --- | --- |
| Step | Variable | SE | *X^2^* | SVR – n (%) | aOR | 95% CI | *p* |
| 1 | Treatment pathway [hospital] |  | 3.04 | 70 (95.9) |  |  |  |
|  | *Drug treatment centres* | .969 | .62 | 32 (94.1) | .47 | .07 – 3.13 | .433 |
|  | *Community pharmacies* | .816 | .253 | 129 (96.3) | .66 | .13 – 3.28 | .615 |
|  | *Needle exchanges* | .770 | 1.25 | 133 (94.3) | .42 | .09 – 1.71 | .263 |
|  | *Nurse-led clinics* | .961 | .10 | 96 (98.0) | 1.34 | .20 – 8.84 | .758 |
|  | *Prison* | .861 | .80 | 76 (95.0) | .463 | .09 – 2.50 | .371 |
|  | Age at treatment | .026 | 2.75 |  | .96 | .91 – 1.01 | .958 |
|  | Constant | 1.50 | 12.81 |  | 212.82 |  | <.001 |
| 2 | Treatment pathway [hospital] |  | 3.23 | 70 (95.9) |  |  |  |
|  | *Drug treatment centres* | .973 | .59 | 32 (94.1) | .47 | .07 – 3.19 | .442 |
|  | *Community pharmacies* | .821 | .21 | 129 (96.3) | .68 | .14 – 3.42 | .684 |
|  | *Needle exchanges* | .775 | 1.29 | 133 (94.3) | .42 | .09 – 1.90 | .415 |
|  | *Nurse-led clinics* | .964 | .09 | 96 (98.0) | 1.34 | .20 – 8.88 | .761 |
|  | *Prison* | .879 | .96 | 76 (95.0) | .42 | .08 – 2.37 | .328 |
|  | Age at treatment | .026 | 3.03 |  | .96 | .91 – 1.01 | .082 |
|  | Sex [male] |  |  | 408 (95.8) |  |  |  |
|  | *Female* | .518 | .35 | 128 (95.5) | .74 | .27 – 2.04 | .557 |
|  | Constant | 1.54 | 13.02 |  | 258.71 |  | <.001 |
| 3 | Treatment pathway [hospital] |  | 2.93 | 70 (95.9) |  |  |  |
|  | *Drug treatment centres* | .974 | .56 | 32 (94.1) | .48 | .07 – 3.26 | .482 |
|  | *Community pharmacies* | .825 | .28 | 129 (96.3) | .65 | .13 – 3.26 | .647 |
|  | *Needle exchanges* | .775 | 1.27 | 133 (94.3) | .42 | .09 – 1.91 | .418 |
|  | *Nurse-led clinics* | .965 | .10 | 96 (98.0) | 1.35 | .20 – 8.84 | .756 |
|  | *Prison* | .897 | .66 | 76 (95.0) | .48 | .08 – 2.80 | .416 |
|  | Age at treatment | .026 | 2.98 |  | .96 | .91 – 1.01 | .085 |
|  | Sex [male] |  |  | 408 (95.8) |  |  |  |
|  | *Female* | .521 | .42 | 128 (95.5) | .71 | .26 – 1.98 | .517 |
|  | Genotype [1] |  | 1.41 | 208 (95.0) |  |  |  |
|  | *2,4,unknown* | .834 | .35 | 22 (91.7) | .61 | .12 – 3.13 | .552 |
|  | *3* | .442 | .73 | 306 (96.5) | 1.46 | .61 – 3.47 | .393 |
|  | Constant | 1.55 | 11.97 |  | 211.14 |  | <.01 |
| 4 | Treatment pathway [hospital] |  | 3.03 | 70 (95.9) |  |  |  |
|  | *Drug treatment centres* | .986 | .62 | 32 (94.1) | .46 | .07 – 3.17 | .429 |
|  | *Community pharmacies* | .833 | .32 | 129 (96.3) | .62 | .12 – 3.18 | .569 |
|  | *Needle exchanges* | .780 | 1.35 | 133 (94.3) | .40 | .09 – 1.87 | .246 |
|  | *Nurse-led clinics* | .964 | .09 | 96 (98.0) | 1.34 | .20 – 8.88 | .760 |
|  | *Prison* | .906 | .72 | 76 (95.0) | .46 | .08 – 2.73 | .395 |
|  | Age at treatment | .026 | 2.60 |  | .96 | .71 – 1.01 | .107 |
|  | Sex [male] |  |  | 408 (95.8) |  |  |  |
|  | *Female* | .522 | .43 | 128 (95.5) | .71 | .26 – 1.98 | .510 |
|  | Genotype [1] |  | 1.42 | 208 (95.0) |  |  |  |
|  | *2,4,unknown* | .442 | .71 | 22 (91.7) | 1.45 | .12 – 3.09 | .542 |
|  | *3* | .442 | .71 | 306 (96.5) | 1.45 | .61 – 3.45 | .398 |
|  | Cirrhosis [yes] |  |  | 50 (94.3) |  |  |  |
|  | *No* | .687 | .13 | 486 (95.9) | 1.28 | .33 – 4.91 | .723 |
|  | Constant | 1.72 | 8.82 |  | 163.01 |  | <.01 |
| **Abbreviations**: ITT, intention-to-treat; SE, standard error; *X^2^*, Wald statistic; SVR, sustained virologic response; aOR, adjusted odds ratio. | | | | | | | |

**Section 3** – full logistic regression tabulation.

Treatment pathway was the predictor of interest for all analyses and was coded for community (i.e. drug treatment centres, pharmacies, needle exchanges, nurse-led community clinics, prisons) or hospital care (referent). In adjusted analyses, age, sex, genotype, and cirrhosis status were included in a stepwise approach. Details of the structure of covariates are in Table 3.1.

| **Table 3.1**: Details of covariates for adjusted analyses. | | | |
| --- | --- | --- | --- |
| **Categorical variable** | **Structure**^a^ | | *df* |
| Treatment pathway | *Hospital care* | | 2 |
|  | Community pathway | |  |
| Age at treatment (n=713) | Mean (95% CI): | 40.35 (39.70 – 40.99) | n/a |
|  | Median: | 39.00 |  |
|  | Min–Max: | 21 – 80 |  |
| Age at treatment (n=630) | Mean (95% CI): | 40.27 (39.58 – 40.96) | n/a |
|  | Median: | 39.00 |  |
|  | Min–Max: | 21 – 80 |  |
| Age at treatment (n=631) | Mean (95% CI): | 40.72 (40.03 – 41.40) | n/a |
|  | Median: | 39.00 |  |
|  | Min–Max: | 21 – 80 |  |
| Age at treatment (n=560) | Mean (95% CI): | 40.64 (39.91 – 41.37) | n/a |
|  | Median: | 39.00 |  |
|  | Min–Max: | 21 – 80 |  |
| Sex | *Male* | | 1 |
|  | Female | |  |
| Genotype | *1* | | 2 |
|  | 2, 4, Unknown | |  |
|  | 3 | |  |
| Cirrhosis | *Cirrhosis* | | 1 |
|  | No cirrhosis | |  |
| ^a^Reference categories are italicised.  **Abbreviations:** df, degrees of freedom; Min, minimum; Max, maximum. | | | |

**Analysis 1**

An unadjusted logistic regression was performed to ascertain the effects of receiving DAA treatment in community pathways compared to the standard hospital pathway on odds of achieving SVR in the intention-to-treat (ITT) group (n=713). This group included treatment completers and non-completers and those with or without an SVR test. Those without an SVR test were assumed treatment failures. The model was not statistically significant, *χ*^2^(1) = 0.01, *p* = .918 and explained <1% (Nagelkerke R^2^) of the variance in SVR. 80.9% of cases were correctly classified. This implies individuals treated through community pathways had no difference in odds of achieving SVR compared to standard hospital care (Table 3.2).

| **Table 3.2**: Results of unadjusted logistic regression in the ITT group, missing SVR assumed failed (n=713). | | | | | | |
| --- | --- | --- | --- | --- | --- | --- |
| Variable | SE | *X^2^* | SVR – n (%) | OR | 95% CI | *p* |
| Hospital care |  |  | 74 (81.3) |  |  |  |
| *Community pathways* | .288 | .010 | 503 (80.9) | .97 | .55 – 1.71 | .919 |
| Constant | .269 | 29.91 |  |  |  | <.001 |
| **Abbreviations**: ITT, intention-to-treat; SE, standard error; *X^2^*, Wald statistic; SVR, sustained virologic response; OR, odds ratio. | | | | | | |

**Analysis 2**

The logistic regression model was adjusted in a stepwise manner in the same ITT group (Table 3.3). Age was assessed for linearity to its log odds and found to be satisfactory. At step 1, the model was not statistically significant, *χ*^2^(2) = .913, *p =* .633 and explained <1% (Nagelkerke R^2^) of the variance in SVR. At step 2, the model remained non-significant, *χ*^2^(3) = 1.84, *p* = .606. At step 3, the model remained non-significant, *χ*^2^(5) = 1.99, *p* = .85. At step 4, the model remained non-significant, *χ*^2^(6) = 1.99, *p* = .921. The amount of variation in SVR explained by the models was unchanged at subsequent steps. Case classification rate (80.9% correct) was unchanged in any step of the adjusted analyses. There were no substantial changes to the parameter estimates or statistical significance of the models at any step in the analysis.

The fully adjusted model implies that in the ITT population, being treated in community settings does not lead to a statistically significant change in the odds of achieving SVR compared to hospital care. This holds true after accounting for *age*, *sex*, *genotype*, and *cirrhosis* status.

| **Table 3.3**: Results of adjusted logistic regressions in the ITT group, missing SVR assumed failed (n=713). | | | | | | | |
| --- | --- | --- | --- | --- | --- | --- | --- |
| Step | Variable | SE | *X^2^* | SVR – n (%) | aOR | 95% CI | *p* |
| 1 | Hospital care |  |  | 74 (81.3) |  |  |  |
|  | *Community pathways* | .313 | .22 | 503 (80.9) | .87 | .47 – 1.60 | .643 |
|  | Age at treatment | .012 | .91 |  | .99 | .97 – 1.01 | .341 |
|  | Constant | .646 | 9.85 |  | 7.58 |  | .002 |
| 2 | Hospital care |  |  | 74 (81.3) |  |  |  |
|  | *Community pathways* | .012 | .213 | 503 (80.9) | .87 | .47 – 1.60 | .865 |
|  | Age at treatment | .012 | 1.20 |  | .99 | .96 – 1.01 | .987 |
|  | Sex [male] |  |  | 431 (81.6) |  |  |  |
|  | *Female* | .216 | .942 | 146 (78.9) | .81 | .53 – 1.24 | .332 |
|  | Constant | .661 | 10.64 |  | 8.65 |  | .001 |
| 3 | Hospital care |  |  | 74 (81.3) |  |  |  |
|  | *Community pathways* | .314 | .21 | 503 (80.9) | .87 | .47 – 1.60 | .644 |
|  | Age at treatment | .012 | 1.17 |  | .99 | .97 – 1.01 | .987 |
|  | Sex [male] |  |  | 431 (81.6) |  |  |  |
|  | *Female* | .217 | .87 | 146 (78.9) | .82 | .53 – 1.25 | .350 |
|  | Genotype [1] |  |  | 228 (81.4) |  |  |  |
|  | *2,4,unknown* | .515 | .06 | 25 (83.3) | 1.13 | .41 – 3.10 | .813 |
|  | *3* | .199 | .06 | 324 (80.4) | .95 | .65 – 1.41 | .812 |
|  | Constant | .668 | 10.60 |  | 8.78 |  | .001 |
| 4 | Hospital care |  |  | 74 (81.3) |  |  |  |
|  | *Community pathways* | .316 | .21 | 503 (80.9) | .87 | .47 – 1.61 | .648 |
|  | Age at treatment | .012 | 1.11 |  | .99 | .96 – 1.01 | .292 |
|  | Sex [male] |  |  | 431 (81.6) |  |  |  |
|  | *Female* | .217 | .87 | 146 (78.9) | .82 | .53 – 1.25 | .350 |
|  | Genotype [1] |  |  | 228 (81.4) |  |  |  |
|  | *2,4,unknown* | .515 | .06 | 25 (83.3) | 1.13 | .41 – 3.01 | .812 |
|  | *3* | .199 | .06 | 324 (80.4) | .95 | .65 – 1.41 | .812 |
|  | Cirrhosis [Yes] |  |  | 52 (80.0) |  |  |  |
|  | *No* | .347 | .00 | 525 (81.0) | .99 | .50 – 1.96 | .978 |
|  | Constant | .784 | 7.76 |  | 8.89 |  | .005 |
| **Abbreviations**: ITT, intention-to-treat; SE, standard error; *X^2^*, Wald statistic; SVR, sustained virologic response; aOR, adjusted odds ratio. | | | | | | | |

**Analysis 3**

An unadjusted logistic regression was performed to ascertain the effects of receiving DAA treatment in community pathways as compared to the standard hospital pathway on odds of achieving SVR in the ITT group who received an SVR test (n=631), i.e. both treatment completers and non-completers were included, but those without an SVR test were excluded. The model was not statistically significant, *χ*^2^(1) = 0.01, *p* = .918 and explained <1% (Nagelkerke R^2^) of the variance in SVR. 91.4% of cases were correctly classified. This implies individuals treated through community pathways had no difference in odds of achieving SVR compared to standard hospital care (Table 3.4).

| **Table 3.4**: Results of unadjusted logistic regression in the ITT group with an SVR test (n=631). | | | | | | |
| --- | --- | --- | --- | --- | --- | --- |
| Variable | SE | *X^2^* | SVR – n (%) | OR | 95% CI | *p* |
| Hospital care |  |  | 74 (91.4) |  |  |  |
| *Community pathways* | .424 | .001 | 503 (91.5) | .97 | .44 – 2.32 | .977 |
| Constant | .395 | 35.56 |  | 10.57 |  | <.001 |
| **Abbreviations**: ITT, intention-to-treat; SE, standard error; *X^2^*, Wald statistic; SVR, sustained virologic response; OR, odds ratio. | | | | | | |

**Analysis 4**

The regression model was adjusted in a stepwise manner in the same ITT group with an SVR test (Table 3.5). Age was assessed for linearity to its log odds and found to be satisfactory.

At step 1, the model was not statistically significant, *χ*^2^(2) = .331, *p* = .847, and explained <1% (Nagelkerke R^2^) of the variance in SVR. At step 2, the model remained non-significant, *χ*^2^(3) = .594, *p* = .898. At step 3, the model remained non-significant, *χ*^2^(5) = 1.11, *p* = .954. At step 4, the model remained non-significant, *χ*^2^(8) = 3.29, *p* = .915. and explained <1% (Nagelkerke R^2^) of the variance in SVR. The amount of variation in SVR explained by the models was unchanged at subsequent steps. Case classification rate (91.4% correct) was unchanged in any step of the adjusted analyses. There were no substantial changes to the parameter estimates or statistical significance of the models at any step in the analysis.

The fully adjusted model has similar implications to the unadjusted analysis: when accounting for age and sex, among those who were followed-up for an SVR test, there was no statistically significant difference in odds of achieving SVR between community pathways and hospital care.

| **Table 3.5**: Results of adjusted logistic regression in the ITT group with an SVR test (n=631). | | | | | | | |
| --- | --- | --- | --- | --- | --- | --- | --- |
| Step | Variable | SE | *X^2^* | SVR – n (%) | aOR | 95% CI | *p* |
| 1 | Hospital care |  |  | 74 (91.4) |  |  |  |
|  | *Community pathways* | .465 | .04 | 503 (91.5) | .91 | .36 – 2.26 | .835 |
|  | Age at treatment | .017 | .33 |  | .99 | .96 – 1.02 | .564 |
|  | Constant | .964 | 8.80 |  | 17.50 |  | .003 |
| 2 | Hospital care |  |  | 74 (91.4) |  |  |  |
|  | *Community pathways* | .466 | .04 | 503 (91.5) | .91 | .36 – 2.26 | .835 |
|  | Age at treatment | .018 | .44 |  | .99 | .96 – 1.02 | .508 |
|  | Sex [male] |  |  | 431 (91.7) |  |  |  |
|  | *Female* | .326 | .27 | 146 (90.7) | .85 | .45 – 1.60 | .605 |
|  | Constant | .991 | 9.00 |  | 19.58 |  | .003 |
| 3 | Hospital care |  |  | 74 (91.4) |  |  |  |
|  | *Community pathways* | .467 | .04 | 503 (91.5) | .91 | .36 – 2.27 | .837 |
|  | Age at treatment | .018 | .47 |  | .99 | .95 – 1.02 | .492 |
|  | Sex [male] |  |  | 431 (91.7) |  |  |  |
|  | *Female* | .327 | .32 | 146 (90.7) | .83 | .44 – 1.58 | .569 |
|  | Genotype [1] |  |  | 228 (90.8) |  |  |  |
|  | *2,4,unknown* | .650 | .78 | 25 (89.3) | .84 | .23 – 2.99 | .783 |
|  | *3* | .295 | .335 | 324 (92.0) | 1.19 | .67 – 2.12 | .563 |
|  | Constant | .995 | 8.55 |  | 18.37 |  | .003 |
| 4 | Hospital care |  |  | 74 (91.4) |  |  |  |
|  | *Community pathways* | .470 | .02 | 503 (91.5) | .94 | .37 – 2.37 | .897 |
|  | Age at treatment | .018 | .66 |  | .99 | 95 – 1.02 | .418 |
|  | Sex [male] |  |  | 431 (91.7) |  |  |  |
|  | *Female* | .327 | .31 | 146 (90.7) | .83 | .44 – 1.58 | .576 |
|  | Genotype [1] |  |  | 228 (90.8) |  |  |  |
|  | *2,4,unknown* | .651 | .07 | 25 (89.3) | .84 | .24 – 3.01 | .791 |
|  | *3* | .295 | .35 | 324 (92.0) | 1.19 | .67 – 2.12 | .557 |
|  | Cirrhosis [Yes] |  |  | 52 (92.9) |  |  |  |
|  | *No* | .570 | .32 | 525 (91.3) | 1.38 | .45 – 4.22 | .572 |
|  | Constant | .998 | 8.77 |  | 19.18 |  | .003 |
| **Abbreviations**: ITT, intention-to-treat; SE, standard error; *X^2^*, Wald statistic; SVR, sustained virologic response; aOR, adjusted odds ratio. | | | | | | | |

**Analysis 5**

An unadjusted logistic regression was performed in the per-protocol (PP) group (n=630). This group included treatment completers with or without an SVR test and assumed those without an SVR test were treatment failures. The model was not statistically significant, *χ*^2^(1) = .04, *p =* .840, and explained <1% (Nagelkerke R^2^) of the variance in SVR. 85.1% of cases were correctly classified. This implies that individuals treated through community pathways had no difference in odds of achieving SVR compared to standard hospital care (Table 3.4).

| **Table 3.6**: Results of unadjusted logistic regression in the PP group, missing SVR assumed failed (n=630). | | | | | | |
| --- | --- | --- | --- | --- | --- | --- |
| Variable | SE | *X^2^* | SVR – n (%) | OR | 95% CI | *p* |
| Hospital care |  |  | 70 (84.3) |  |  |  |
| *Community pathways* | .325 | .04 | 466 (85.2) | 1.07 | .57 – 2.02 | .839 |
| Constant | .302 | 31.08 |  | 5.39 |  | <.0001 |
| **Abbreviations**: ITT, intention-to-treat; SE, standard error; *X^2^*, Wald statistic; SVR, sustained virologic response; OR, odds ratio. | | | | | | |

**Analysis 6**

The logistic regression model was adjusted in a stepwise approach for the same PP group (Table 3.7). Age was assessed for linearity to its log odds and found to be satisfactory.

At step 1, the model was not statistically significant, *χ*^2^(2) = 1.70, *p =* .428 and explained <1% of the variation in SVR (Nagelkerke R^2^). At step 2, the model remained non-significant, *χ*^2^(3) = 3.17, *p* = .367. At step 3, the model remained non-significant, *χ*^2^(5) = 3.21, *p* = .667. At step 4, the model remained non-significant, *χ*^2^(6) = 3.22, *p* = .781. The amount of variation in SVR explained by the models was unchanged at subsequent steps. Case classification rate (85.1% correct) was unchanged in any step of the adjusted analyses. There were no substantial changes to the parameter estimates or statistical significance of the models at any step in the analysis.

The fully adjusted model has similar implications to the unadjusted analysis: when accounting for age and sex, among those who completed treatment, with or without an SVR test, there was no statistically significant difference in odds of achieving SVR between community pathways and hospital care in those who completed treatment.

| **Table 3.7**: Results of adjusted logistic regressions in the PP group, missing SVR assumed failed (n = 630). | | | | | | | |
| --- | --- | --- | --- | --- | --- | --- | --- |
| Step | Variable | SE | *X^2^* | SVR – n (%) | aOR | 95% CI | *p* |
| 1 | Hospital care |  |  | 70 (84.3) |  |  |  |
|  | *Community pathways* | .360 | .13 | 466 (85.2) | .88 | .44 – 1.78 | .722 |
|  | Age at treatment | .014 | 1.67 |  | .98 | .96 – 1.01 | .196 |
|  | Constant | .769 | 11.33 |  | 13.30 |  | .001 |
| 2 | Hospital care |  |  | 70 (84.3) |  |  |  |
|  | *Community pathways* | .361 | .13 | 466 (85.2) | .88 | .43 – 1.78 | .722 |
|  | Age at treatment | .014 | 2.13 |  | .98 | .95 – 1.01 | .145 |
|  | Sex [male] |  |  | 408 (85.9) |  |  |  |
|  | *Female* | .254 | 1.51 | 128 (82.6) | .73 | .45 – 1.20 | .219 |
|  | Constant | .787 | 12.42 |  | 16.02 |  | <.0001 |
| 3 | Hospital care |  |  | 70 (84.3) |  |  |  |
|  | *Community pathways* | .361 | .12 | 466 (85.2) | .88 | .43 – 1.79 | .727 |
|  | Age at treatment | .014 | 2.11 |  | .98 | .95 – 1.01 | .147 |
|  | Sex [male] |  |  | 408 (85.9) |  |  |  |
|  | *Female* | .255 | 1.49 | 128 (82.6) | .73 | .44 – 1.21 | .223 |
|  | Genotype [1] |  |  | 208 (85.6) |  |  |  |
|  | *2,4,unknown* | .576 | .03 | 22 (84.6) | .90 | .29 – 2.79 | .855 |
|  | *3* | .236 | .03 | 306 (84.8) | .96 | .61 – 1.53 | .869 |
|  | Constant | .796 | 12.33 |  | 16.37 |  | <.0001 |
| 4 | Hospital care |  |  | 70 (84.3) |  |  |  |
|  | *Community pathways* | .363 | .12 | 466 (85.2) | .88 | .43 – 1.79 | .725 |
|  | Age at treatment | .014 | 1.95 |  | .98 | .95 – 1.01 | .162 |
|  | Sex [male] |  |  | 408 (85.9) |  |  |  |
|  | *Female* | .255 | 1.49 | 128 (82.6) | .73 | .44 – 1.21 | .223 |
|  | Genotype [1] |  |  | 208 (85.6) |  |  |  |
|  | *2,4,unknown* | .577 | .03 | 22 (84.6) | .90 | .29 – 2.79 | .855 |
|  | *3* | .236 | .03 | 306 (84.8) | .96 | .61 – 1.53 | .868 |
|  | Cirrhosis [Yes] |  |  | 50 (83.3) |  |  |  |
|  | *No* | .388 | .00 | 486 (85.3) | .98 | .46 – 2.10 | .960 |
|  | Constant | .799 | 12.22 |  | 16.32 |  | <.0001 |
| **Abbreviations**: ITT, intention-to-treat; SE, standard error; *X^2^*, Wald statistic; SVR, sustained virologic response; aOR, adjusted odds ratio. | | | | | | | |

**Analysis 7**

An unadjusted logistic regression was performed in the per-protocol (PP) group who received an SVR test (n=560). The model was not statistically significant, *χ*^2^(1) = .01, *p =* .936, and explained <1% (Nagelkerke R^2^) of the variance in SVR. 95.7% of cases were correctly classified. Individuals treated through community pathways had no difference in odds of achieving SVR compared to standard hospital care (Table 3.4).

| **Table 3.8**: Results of unadjusted logistic regression in the PP group with an SVR test (n=560). | | | | | | |
| --- | --- | --- | --- | --- | --- | --- |
| Variable | SE | *X^2^* | SVR – n (%) | OR | 95% CI | *p* |
| Hospital care |  |  | 70 (95.9) |  |  |  |
| *Community pathways* | .630 | .01 | 466 (95.7) | .95 | .28 – 3.27 | .937 |
| Constant | .590 | 28.54 |  | 23.33 |  | <.0001 |
| **Abbreviations**: ITT, intention-to-treat; SE, standard error; *X^2^*, Wald statistic; SVR, sustained virologic response; OR, odds ratio. | | | | | | |

**Analysis 8**

The logistic regression model was adjusted in a stepwise approach for the same PP group (Table 3.9). Age was assessed for linearity to its log odds and found to be satisfactory.

At step 1, the model was not statistically significant, *χ*^2^(2) = 2.32, *p =* .314 and explained <1% of the variance in SVR (Nagelkerke R^2^). At step 2, the model remained non-significant, *χ*^2^(3) = 2.45, *p* = .484. At step 3, the model remained non-significant, *χ*^2^(5) = 4.10, *p* = .535. At step 4, the model remained non-significant, *χ*^2^(6) = 5.12, *p* = .660. The amount of variation in SVR explained by the models was unchanged at subsequent steps. Case classification rate (95.7% correct) was unchanged in any step of the adjusted analyses. There were no substantial changes to the parameter estimates or statistical significance of the models at any step in the analysis.

The fully adjusted model has similar implications to the unadjusted analysis: when accounting for age and sex, among those who completed treatment, there was no statistically significant difference in odds of achieving SVR between community pathways and hospital care in those who completed treatment and received an SVR test.

| **Table 3.9**: Results of adjusted logistic regression in the PP group with an SVR test (n=560). | | | | | | | |
| --- | --- | --- | --- | --- | --- | --- | --- |
| Step | Variable | SE | *X^2^* | SVR – n (%) | aOR | 95% CI | *p* |
| 1 | Hospital care |  |  | 70 (95.9) |  |  |  |
|  | *Community pathways* | .698 | .51 | 466 (95.7) | .61 | .16 – 2.39 | .477 |
|  | Age at treatment | .025 | 2.51 |  | .96 | .92 – 1.01 | .121 |
|  | Constant | 1.443 | 12.66 |  | 170.03 |  | <.0001 |
| 2 | Hospital care |  |  | 70 (95.9) |  |  |  |
|  | *Community pathways* | .700 | .51 | 466 (95.7) | .61 | .15 – 2.39 | .476 |
|  | Age at treatment | .025 | 2.54 |  | .96 | .92 – 1.01 | .961 |
|  | Sex [male] |  |  | 408 (95.8) |  |  |  |
|  | *Female* | .491 | .14 | 128 (95.5) | .83 | .32 – 2.18 | .711 |
|  | Constant | 1.475 | 12.63 |  | 189.17 |  | <.0001 |
| 3 | Hospital care |  |  | 70 (95.9) |  |  |  |
|  | *Community pathways* | .701 | .49 | 466 (95.7) | .61 | .16 – 2.42 | .483 |
|  | Age at treatment | .02 | 2.60 |  | .78 | .30 – 2.06 | .618 |
|  | Sex [male] |  |  | 408 (95.8) |  |  |  |
|  | *Female* | .495 | .25 | 128 (95.5) | .78 | .30 – 2.06 | .618 |
|  | Genotype [1] |  |  | 208 (95.0) |  |  |  |
|  | *2,4,unknown* | .808 | .42 | 22 (91.7) | .59 | .12 – 2.88 | .515 |
|  | *3* | .438 | .84 | 306 (96.5) | 1.51 | .64 – 3.55 | .350 |
|  | Constant | 1.47 | 11.78 |  | 155.51 |  | .001 |
| 4 | Hospital care |  |  | 70 (95.9) |  |  |  |
|  | *Community pathways* | .705 | .51 | 466 (95.7) | .61 | .15 – 2.41 | .476 |
|  | Age at treatment | .025 | 2.30 |  | .96 | .92 – 1.01 | .130 |
|  | Sex [male] |  |  | 408 (95.8) |  |  |  |
|  | *Female* | .496 | .25 | 128 (95.5) | .78 | .30 – 2.06 | .616 |
|  | Genotype [1] |  |  | 208 (95.0) |  |  |  |
|  | *2,4,unknown* | .808 | .43 | 22 (91.7) | .59 | .12 – 2.88 | .515 |
|  | *3* | .438 | .87 | 306 (96.5) | 1.51 | .64 – 3.55 | .351 |
|  | Cirrhosis [Yes] |  |  | 50 (94.3) |  |  |  |
|  | *No* | .675 | .02 | 486 (95.9) | .90 | .24 – 3.39 | .878 |
|  | Constant | 1.474 | 11.66 |  | 153.18 |  | .001 |
| **Abbreviations**: ITT, intention-to-treat; SE, standard error; *X^2^*, Wald statistic; SVR, sustained virologic response; aOR, adjusted odds ratio. | | | | | | | |
